# Estimating health system opportunity costs: the role of non-linearities and inefficiency

**DOI:** 10.1186/s12962-022-00391-y

**Published:** 2022-10-29

**Authors:** Karla Hernandez-Villafuerte, Bernarda Zamora, Yan Feng, David Parkin, Nancy Devlin, Adrian Towse

**Affiliations:** 1 German Cancer Research Center (DKFZ), Heidelberg, Germany; 2 Imperial College London, London, UK; 3 Queen Mary University of London, London, UK; 4 Office of Health Economics, London, UK; 5 University of Melbourne, Melbourne, Australia

**Keywords:** Opportunity cost, Cost-effectiveness threshold, Quantile regression, DEA, Outcome elasticities, English NHS

## Abstract

**Background:**

Empirical estimates of health system opportunity costs have been suggested as a basis for the cost-effectiveness threshold to use in Health Technology Assessment. Econometric methods have been used to estimate these in several countries based on data on spending and mortality. This study examines empirical evidence on four issues: non-linearity of the relationship between spending and mortality; the inclusion of outcomes other than mortality; variation in the efficiency with which expenditures generate health outcomes; and the relationship among efficiency, mortality rates and outcome elasticities.

**Methods:**

Quantile Regression is used to examine non-linearities in the relationship between mortality and health expenditures along the mortality distribution. Data Envelopment Analysis extends the approach, using multiple measures of health outcomes to measure efficiency. These are applied to health expenditure data from 151 geographical units (Primary Care Trusts) of the National Health Service in England, across eight different clinical areas (Programme Budget Categories), for 3 fiscal years from 2010/11 to 2012/13.

**Results:**

The results suggest differences in efficiency levels across geographical units and clinical areas as to how health resources generate outcomes, which indicates the capacity to adjust to a decrease in health expenditure without affecting health outcomes. Moreover, efficient units have lower absolute levels of mortality elasticity to health expenditure than inefficient ones.

**Conclusions:**

The policy of adopting thresholds based on estimates of a single system-wide cost-effectiveness threshold assumes a relationship between expenditure and health outcomes that generates an opportunity cost estimate which applies to the whole system. Our evidence of variations in that relationship and therefore in opportunity costs suggests that adopting a single threshold may exacerbate the efficiency and equity concerns that such thresholds are designed to counter. In most health care systems, many decisions about provision are not made centrally. Our analytical approach to understanding variability in opportunity cost can help policy makers target efficiency improvements and set realistic targets for local and clinical area health improvements from increased expenditure.

**Supplementary Information:**

The online version contains supplementary material available at 10.1186/s12962-022-00391-y.

## Background

Providing health care has an opportunity cost. In health care systems with a fixed budget this is the health benefits forgone from other health care that could have been provided with the resources used. This should be fundamental to many health policy considerations, including efficiency improvement and sociodemographic and geographical equity. Recent work quantifying health system opportunity costs [1–10] has focussed on the adoption of new technologies and their displacement impact on other health care, usually expressed as the search for a ‘threshold’ against which Health Technology Assessment (HTA) agencies can judge cost-effectiveness.

Martin et al. [1, 2] developed methods for examining the impact of health expenditure on health which were used by Claxton et al. [3] to estimate an opportunity cost based threshold for the NHS in England. This was updated by Lomas et al. [4] and has recently been revisited by Martin et al. [5]. Estimates have now been published for several countries [6–10]. Although these studies use slightly different approaches, most follow Claxton et al*.* in applying econometric methods to health system data to examine the relationship between health care expenditures and health outcomes from variations observed across health care ‘programmes’ and administrative units (health care payers or commissioners). They estimate the average relationship between spending and outcomes, based on mortality converted to Quality Adjusted Life Years (QALYs). The England NHS studies calculate QALYs as an adjustment to mortality figures, rather than measuring morbidity as a separately sourced category of health gain.

This paper addresses three issues that have been raised about these methods [11]: first, linear regression models may not correctly specify the relationship between expenditure and mortality; secondly, using mortality (or QALYs) as the only health care outcome may not fully reflect health system priorities; and thirdly, variations in the efficiency with which health is produced may impact on the observed relationship of inputs and outcomes. We applied Quantile Regression (QR) and Data Envelopment Analysis (DEA) to English NHS data. QR permits estimation of non-linear relationships, examining point estimates of the expenditure/mortality relationship at different parts of the mortality distribution to show differences across PCTs with low to high mortality rates. DEA permits inclusion of non-mortality health outcomes aligned with health system priorities and enables measurement of the variations in efficiency. Use of these methods allowed us to address a fourth issue: the relationship among efficiency, mortality rates and outcome elasticities.

## Methods

### Quantile regression

QR was used to explore differences across the expenditure/outcome relationship as between 151 local commissioners of NHS health care in England, focussing on understanding differences as between those with low and high mortality rates in different clinical areas (detail on the included periods and variables considered is presented in "Data" section).

In classical linear regression, the estimated covariate effects are the same across the data distribution. QR provides a more complete picture of covariate effects by estimating a family of conditional quantile functions [12]. It estimates a point at any part of the distribution, without splitting the sample into different groups. Different quantiles are obtained by minimising a sum of asymmetrically weighted absolute residuals, with the median (0.5 quantile or 50th percentile) obtained by minimising the unweighted absolute value. Other quantiles use weights, for example, we can estimate the 0.75 quantile (75th percentile) which leaves ¾ of observations below and ¼ above the quantile. The point estimate of this conditional quantile can be obtained by minimising the sum of absolute residuals but penalising underpredictions more than overpredictions, where the weight assigned to underpredictions (0.75) represents the quantile. Classical linear regression models have a unique slope coefficient, which in this case represents the percentage impact on mortality of marginal changes in expenditure, hereafter referred to as *mortality elasticity*. QR estimates a slope coefficient, and therefore a different mortality elasticity estimate, for each quantile by introducing different weights at different points of the outcome distribution.

For comparability with the estimates published by Lomas et al. [4], we used the same specification for the QR model as their linear regression model: an outcome function linking mortality and expenditure in a clinical area, including covariates correlated with expenditure and mortality, such as different health needs due to demographic composition and socioeconomic factors. The estimator is:

1 $${Q}_{h}\left({\tau }_{i}|{n}_{ij}, {x}_{ij}\right)={\alpha }_{j}\left({\tau }_{i}\right)+{\beta }_{j}\left({\tau }_{i}\right){x}_{ij}+{\gamma }_{j}\left({\tau }_{i}\right){n}_{ij}+{w}_{ij}$$

where ${Q}_{h}\left({\tau }_{i}|{n}_{ij}, {x}_{ij}\right)$ is the $\tau$^th^ quantile on mortality rate *h* for each commissioner* i* in each clinical area *j*, conditional on health care need *n*_*ij*_ and local health expenditure per head *x*_*ij*_. The random error *w*_*ij*_ is allowed to be correlated with *x*_*ij*_ to consider endogeneity of health expenditure. The effect of expenditure on mortality is measured by $\beta$, which can be assessed at any point $\tau$ of the mortality distribution in the range (0, 1). Edney et al*.* have estimated a similar QR model of mortality reduction for Australia [13].

There are 151 quantiles for each clinical area equation, each of which, $\tau$_i_, represents a different local commissioner ranked according to Standardised Years of Life Lost Rate (SYLLR). Within each clinical area, the first quantile is therefore 1/151, for the commissioner with the largest SYLLR, and 151/151 = 1 for the one with the smallest SYLLR.

QR produces more robust estimations in the presence of non-normally distributed errors and outliers and preserves the conditional quantiles in transformations of the variables such as the logarithmic.

Our method accounts for the potential endogeneity of expenditure by commissioner which may result, for example, from poorer health outcome areas getting more funding; health expenditures per person are adjusted according to population needs measured by the “unified weighted population index” [14]. Our Instrumental Variables (IVs) are socioeconomic variables justifiable on an empirical basis and related to IVs proposed on theoretical grounds, for example as part of the funding rule used to allocate health budgets across local authorities [15, 16]. The exogeneity and validation tests of IVs were performed using Generalized Method of Moments (GMM) estimation, the results of which are in given in Additional file 2 Supplementary Material: Tables S2 to S7. However, GMM estimates are sensitive to the number of near redundant instruments which produce a finite sample bias towards underestimation of the mortality elasticity in a similar sample of England NHS local commissioners [15]. We used GMM estimation in four models, using two IVs in two models, and three and four IVs for the other two models, so the test of overidentifying restrictions and GMM estimation of the elasticity is unlikely to be affected by redundant instruments.

Where the exogeneity hypothesis was not rejected, we estimated the conditional mean of the mortality distribution by Ordinary Least Squares (OLS) and the mortality elasticity at different quantiles using a simple QR model. Where there is evidence of endogeneity, the QR model accounts for this. The method is a two-step generated regressors approach, proposed in the context of QR for recursive structural equation models by Ma and Koenker [17] and recently extended by Chen et al. [18]. To compare for robustness, we also tested in one PBC the control function approach, a similar two-stage method proposed by Chernozhukov et al. [19], resulting in almost identical mortality elasticities at different quantiles for Cancer.

The first of the two stages is the IV two stage least squares (2SLS) estimator and the second is a system of QR models where the joint variance and covariance of the system are estimated by bootstrap, accounting for adjustment of the measurement error in generated regressors, and improving the robustness of inference in small samples.

### Data envelopment analysis

DEA is a linear programming-based method that establishes a best-practice production frontier in which each production unit’s efficiency can be judged against the performance of similar units [20]. In this case, the production units are local commissioners. DEA does not assume a specific functional form for the production function that underlies the frontier and allows analysis of multiple inputs and outputs. It therefore allows us to include more than one health outcome in addition to or replacing mortality in analysing the relationship between expenditure and health outcomes. The estimate of the relative efficiency of each commissioner is in effect the potential that they have to change expenditure in a clinical area without affecting health outcomes. ‘Input oriented’ DEA allows us to observe how much the inputs (in our case healthcare expenditures) of less efficient commissioners could in principle be decreased without affecting outcomes. The opportunity cost of funding a new health technology in terms of health outcomes will be lower if it is possible to release funds by improving the efficiency with which existing services are provided.

DEA constructs a measure of technical efficiency based on the distance between composite inputs and composite outputs. It identifies the most efficient commissioners, those that achieve the highest level of health outcomes at a given expenditure, which form the production frontier. An efficiency score is obtained for each commissioner, where full efficiency = 1 and < 1 means it operates at less than best practice efficiency, below the frontier.

**Fig. 1:**
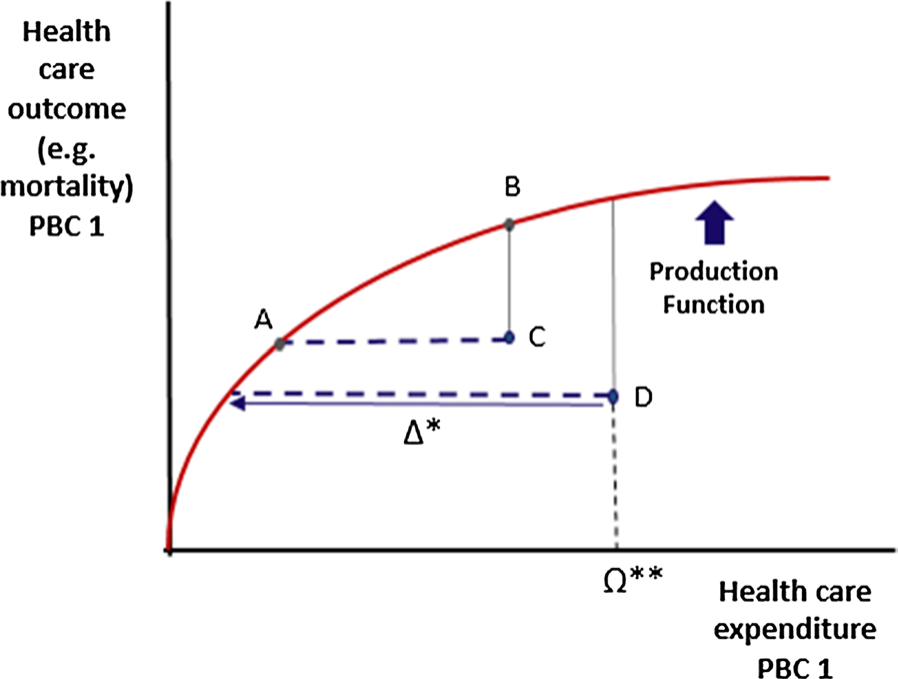
Production function identifying efficient and inefficient PCTs

Figure 1 illustrates efficiency scores and the possible decrease in expenditure that a commissioner could achieve without affecting outcomes in a particular clinical area. A and B represent efficient commissioners who would reduce health outcomes if they spent less; C and D represent inefficient commissioners who could reorganise their production of health to achieve the same outcomes with lower expenditure, that is without incurring opportunity costs. The expenditure reduction by commissioner D (∆*) would improve efficiency without affecting health outcomes. The ratio of ∆* to Ω** shows the proportion of current expenditure that could be reduced without affecting outcomes.

If there are economies of scale in health production, then the size of the unit will influence efficiency. If present, this needs to be adjusted for, in order to focus on technical efficiency and inefficiency. We used Simar and Wilson's returns-to-scale test for input-oriented DEA to guide the choice of model, which tests a constant returns to scale (CRS) assumption against the alternative of variable returns to scale (VRS), using the ratio of means [21] and the mean of ratios less one [22]. The Kruskal–Wallis rank test examined frontier shifts between CRS and VRS.

DEA efficiency scores are sensitive to outliers. The Bogetoft and Otto test [23] was applied to identify outliers. Commissioners with a test statistic below 0.975 were considered outliers and excluded from the estimation.

As noted above, DEA may include environmental variables (EVs), which are exogenous factors that impact outputs (health outcomes) but are not under the control of the commissioners. Commonly used methods to consider EVs in DEA have two problems: prior assumptions about the direction of the effects are needed and estimated efficiency scores cannot be directly linked to the efficiency frontier. We avoid these following the Fried et al. three-stage procedure [24]. First, DEA is applied to health outcomes and inputs only, to identify outliers and obtain initial measures of commissioners’ performance. Secondly, stochastic frontier analysis (SFA) [20] is used to regress first stage performance measures against selected EVs. This provides, for each input, a three-way decomposition of performance variation into that attributable to EV effects, inefficiency and statistical noise. Thirdly, inputs are adjusted to account for the impact of the EVs effects and the statistical noise uncovered in the second stage, and DEA is used to re-evaluate commissioners’ efficiency.

We apply three exclusion criteria for outcome variables: more than 20% of data missing; intermediate rather than final outcome; and the outcome is less important for the estimation of the efficiency scores, according to a Kolmogorov–Smirnov test, than a second outcome with which it is highly correlated (R >  = 0.5).

### Comparing DEA and QR

DEA and QR explore health system efficiency from different perspectives. QR estimates parametrically the mortality elasticity assuming an underlying production function, while DEA estimates non-parametric efficiency scores for all units at or outside the production function. We used two comparison methods to assess the consistency of QR and DEA findings and what they tell us in combination about the efficiency of expenditure at the margin: Spearman rank correlation to represent the sign of the pairwise correlation between the efficiency score and the absolute value of the mortality elasticity; and a t-test comparing the mean mortality elasticities between efficient and inefficient commissioners.

## Data

We used publicly available sources accessible though NHS Digital (previous NHS Indicators Portal) and from NHS Programme Budget Categories expenditure data. The geographical units of analysis are Primary Care Trusts (PCTs), which were at that time local commissioners of NHS health care in England. PCT expenditures are available for those years subdivided by Programme Budgeting Category (PBC), a post hoc allocation of spending to different clinical areas developed by the NHS, but no longer published [25]. We used PBC data relating to eight clinical areas: Infectious Disease, Cancer, Endocrine, Mental Health, Circulation, Respiratory, Gastrointestinal and Maternity (Table 1). Expenditures were adjusted using the UK Department of Health’s Need Index, which adjusts for the health care needs of each PCT’s population in addition to those due to age, and its Market Forces Factor, which accounts for unavoidable geographical variations in the costs of providing services [14]. After 2013, the NHS restructured local commissioning from PCTs to Clinical Commissioning Groups (CCGs) with a large part (about 30%) of the NHS budget retained as a central budget for specialised services.

**Table 1:** Main variables included in the DEA and the QR analysis

	Median	Mean	Min.	1st Qu	3rd Qu	Max	Std. Dev.
*Expenditure per person (£) in each PBC adjusted by need and price*							
PBC 1 Infectious Diseases							
2012/13	24.6	30.8	12.2	19.7	35.3	99.1	17.5
PBC 2 Cancer							
2010/11	103.7	104.3	60.6	89.7	116.4	193.3	20.6
2011/12	104.2	104.2	55.3	90.5	116.3	161.8	17.4
2012/13	105.7	106.4	49.4	91.2	118.6	165.5	20.1
PBC 4 Endocrine							
2010/11	53.2	54.3	38.4	48.6	59.1	86.1	8.4
2011/12	54.4	56.0	42.2	51.1	59.7	88.1	7.7
2012/13	56.9	57.7	40.7	52.5	62.1	79.6	7.2
PBC 5 Mental Health							
2010/11	203.0	214.0	48.0	182.0	230.0	447.0	56.2
2011/12	207.0	215.0	121.0	184.0	232.0	409.0	47.9
2012/13	209.0	217.0	143.0	188.0	236.0	412.0	46.1
PBC 10 Circulation							
2010/11	130.9	132.4	87.8	119.7	146.0	215.2	20.5
2011/22	130.7	130.5	86.6	118.0	143.5	168.6	17.3
2012/13	126.9	128.3	82.5	115.2	140.8	175.0	18.3
PBC 11 Respiratory							
2010/11	82.4	82.5	48.9	75.6	88.7	123.0	11.7
2011/12	83.7	84.4	55.7	78.1	88.7	125.2	10.2
2012/13	89.4	89.1	55.7	81.9	94.8	121.9	10.7
PBC 13 Gastrointestinal							
2010/11	84.7	84.3	34.8	77.5	92.5	140.0	14.4
2011/12	86.6	87.3	56.2	80.5	94.3	118.7	11.1
2012/13	89.2	89.4	60.1	82.2	95.8	117.7	11.1
PBC 18 Maternity							
2010/11	64.1	69.9	32.2	54.3	79.8	167.9	22.1
2011/12	65.0	69.3	34.7	56.0	77.0	168.8	20.2
2012/13	62.1	66.7	35.1	54.1	76.0	162.8	20.1
Healthy individuals (PBC 21)							
2010/11	41.7	42.6	0.03	30.1	53.6	122.3	21.7
2011/12	39.6	39.5	0.17	28.9	49.8	88.4	16.1
2012/13	35.5	36.1	2.22	26.5	47.3	99.2	16.9
Social care needs (PBC22)							
2010/11	41.0	55.5	0.26	21.5	65.2	488.0	71.6
2011/12	53.9	60.6	0.29	31.8	71.7	429.8	56.3
2012/13	63.4	69.1	1.83	40.3	86.2	412.4	50.0
Other areas of spend (PBC 23)							
2010/11	284.6	305.0	211.5	257.7	319.8	719.4	79.6
2011/12	301.1	308.0	217.7	273.0	336.4	469.5	49.4
2012/13	312.0	319.6	220.0	274.4	351.9	516.2	57.0
*Standardised years of life lost rate, average 2012–2014*							
Infectious	5.6	6.4	2.4	4.4	8.1	14.4	2.6
Cancer	159.8	162.1	125.1	146.8	175.1	207.9	19.5
Endocrine	4.0	4.2	0.7	3.0	5.2	9.3	1.6
Circulation	86.7	88.9	50.2	74.9	102.2	141.8	18.7
Respiratory	23.7	25.6	12.3	18.9	31.1	57.5	8.7
Gastrointestinal	22.4	24.4	10.2	16.5	30.1	61.8	9.5

Sources: Based on data from the “Exposition book” elaborated by the Department of Health (DH, 2013)

Available at https://www.networks.nhs.uk/nhs-networks/health-investment-network/news/2012-13-programme-budgeting-data-is-now-available

Mortality statistics from NHS Digital Compendium Indicators (Office of National Statistics). Data from Local Authorities mapped to PCTs

1. The Infectious diseases PBC is only estimated in the QR model, with explanatory variable 2012/13

2. PBC 21, PBC 22, and PBC 23 are considered general spend PBCs since they can be related to all clinical areas

3. The DEA general spend variable is the sum of PBC 21, PBC 22 and PBC 23 expenditures, except for Cancer, which includes only PBC 21 and PBC 22, and Circulation, which only includes PBC 21

Mortality is measured as the SYLLR for those under 75 years, averaged over 2012–2014 and standardised using the 2013 European Standard Population (Table 1). The QR analysis only has a single outcome indicator, mortality. This is not the main outcome indicator for either Maternity or Mental Health, so these two were only included in the DEA analysis. The QR analysis included covariates to control for confounding effects on outcomes (Table 2). Other QR variables affect health outcomes only via its effect on expenditure; these are used as instrumental variables (IVs) to correct for endogeneity bias.

**Table 2:** Pool of indicators used as environmental, exogenous, and instrumental variables

	Mean	Std. dev.	Min.	Max.
Deprivation variables				
Index of Multiple Deprivation (IMD)	23.6	8.41	8.81	45.31
IMD Income Scale	49,791	22,864	14,110	122,060
IMD Employment Scale	19,902	9332	5,000	54,350
Proportion most deprived areas	0.24	0.18	0.00	0.77
Distance to target 2010/11	0.00	1.00	− 1.15	3.95
Health Need variables				
Combining Age Related and Additional Needs (CARAN) index	1.025	0.129	0.727	1.354
HIV prevention Index	1.080	0.666	0.564	4.098
Socioeconomic variables				
OWNOCC: % of households that are owner occupied	0.615	0.116	0.242	0.754
LAHRENT: % of households that are rented from LA or HA	0.188	0.073	0.081	0.437
PRIVRENT: % of households that are rented from private landlords	0.162	0.060	0.084	0.376
NQUAL all: % of population with no qualifications	0.230	0.051	0.101	0.352
PROFOCCU: % of those aged 16–74 years in managerial and professional occupations	0.305	0.069	0.181	0.547
LONE 65 and over: % of households that are one person 65 and over households	0.122	0.021	0.060	0.167
LONEPARH: % of households that are lone parent households with dependent children	0.075	0.017	0.047	0.144
POPPUCAR: % of population providing unpaid care	0.102	0.014	0.065	0.126
POPPUCAR1: % of population providing unpaid care for 1–19 h a week	0.063	0.009	0.043	0.081
POPPUCAR2: % of population providing unpaid care for 20–49 h per week	0.014	0.003	0.009	0.022
POPPUCAR3: % of population providing unpaid care for > 50 h a week	0.024	0.006	0.012	0.040
POPALLLTI: % of population with LTI/disability	0.180	0.032	0.112	0.256
POP16_64LTI: % of population of working age with LTI/disability aged 16–74 years	0.133	0.027	0.076	0.206
HHNOCAR: % of households without a car	0.284	0.118	0.126	0.648
BORNEXEU: Residents born outside the EU divided by all residents	0.103	0.100	0.012	0.424
WHITEEG: Population in white ethnic group divided by total population	0.837	0.166	0.290	0.985
PC74LTUN: % of those aged 16–74 years that are long-term unemployed	0.019	0.006	0.010	0.037
FTSTUDEN: % of population aged 16–74 years that are full-time students	0.094	0.037	0.056	0.226

Sources: English indices of deprivation 2010

https://www.gov.uk/government/statistics/english-indices-of-deprivation-2010,

Census 2011

https://www.nomisweb.co.uk/census/2011

The DEA included health outcome indicators relating to four of the five priority domains explicitly identified by NHS England in the NHS Five Year Forward View [26]. (The fifth domain ‘safe environment’ does not have measurable outcomes.)

Table 3 shows the outcomes additional to SYLLR included in the DEA. Some data were transformed to meet the requirement that outputs take positive values, for example the inverse of SYLLR was used so that extra expenditure inputs generate extra outputs. 2014 outcome data were used to account for the lag between expenditures and their effect on outcomes. Infectious disease was excluded from the DEA because no outcome data apart from mortality are publicly available.

**Table 3:** Summary statistics of other outcome measures included in DEA

	Median	Mean	Min	1st Qu	3rd Qu	Max	Missing	Std Dev
Cancer								
OneYSurv_2014^1^	68.7	67.1	17.6	66.9	70.0	73.6	0	6.8
Endocrine								
DiabComplications_2014_INV^1^	1.0	1.1	0.6	0.9	1.2	6.6	0	0.5
Mental Health								
SMH/CPA_Independently^1^	63.8	60.6	1.6	50.9	73.5	92.6	0	19.3
SMH/CPA_Employment^1^	6.0	6.6	0.1	4.2	8.2	19.9	2	3.4
ExcessMort_2014_INV^2^	0.29	0.3	0.17	0.25	0.33	1.1	0	0.1
MH_HRQoL_2014^1^	0.5	0.5	0.3	0.5	0.6	0.6	0	0.1
Circulation								
CardiacRehab_2014^1^	0.3	0.3	0.0	0.2	0.4	0.7	23	0.2
Stroke_discharge_2014^1^	0.8	0.7	0.0	0.5	0.9	1.0	2	0.2
Respiratory								
Emergency_Child_2014_INV^1^	0.3	0.3	0.2	0.2	0.4	1.3	1	0.2
Gastrointestinal								
AlcoholLiverEmerg_2014_INV^1^	4.4	5.0	1.7	3.0	5.9	32.9	0	3.7
Maternity								
NeonatalMort_2014_INV^2^	14.8	15.4	7.7	12.3	17.2	30.9	1	4.5
MAT01_Point_2012^3^	288.0	319.0	72.0	210.0	390.0	870.0	0	153.0

Sources: NHS Digital. ^1^CCG Outcomes Framework Indicator Set for year 2014 data mapped from CCGs to PCTs. ^2^NHS Outcomes Frameworks Indicators for 2014 mapped from CCGs/Local Authorities to PCTs. ^3^Quality Outcomes Framework available at PCT level

INV: Inverse of the variable used. *OneYSurv*: One-year net survival for adults (15–99) diagnosed with cancer. *DiabComplications*: Indirectly age and sex standardised ratio of complications in people with diabetes. SMH/CPA: % of working age adults (18–69) who are receiving secondary mental health services and who are on the Care Programme Approach at the end of the month. *SMH/CPA _Independently*: SMH/CPA who are recorded as living independently (with or without support). *SMH/CPA_Employment*: SMH/CPA who are recorded as being employed. *ExcessMort*: Excess under 75 mortality rate in adults with serious mental illness (standardised mortality ratio expressed as a percentage based on general population and mental health population mortality rates). *MH_HRQoL*: Directly standardised average health-status (EQ-5D) score for individuals with long-term mental health condition. *CardiacRehab*: % of referrals to a cardiac rehabilitation programme that were recorded as completed within 365 days of the start of an associated hospital admission. *Stroke_discharge*: People with stroke who are discharged from hospital. *Emergency_Child*: Directly age and sex standardised admission rate for emergency admissions for children aged 18 years and under with lower respiratory tract infections per 100,000 registered patients. *AlcoholLiverEmerg*: Directly age and sex standardised rate of emergency admissions for alcohol related liver disease in adults aged 19 years and older, per 100,000 registered patients. *NeonatalMort*: Neonatal mortality and stillbirths (Directly age-standardised rates). *MAT01_Point*: Maternity Services Quality and Outcomes Framework (QOF)

As a result of the exclusions, five of the eight clinical areas could be analysed using both QR and DEA.

The DEA includes two ‘environmental variables’ (EVs): the Index of Multiple Deprivation (IMD) and the ‘distance to target’ (Table 2). The IMD measures socioeconomic differences for local populations [27, 28]. “Distance to target” measures the difference between a PCT’s actual funding allocation, and the funding required, according to the NHS allocation formula [14], to meet local populations’ health needs. The DEA model assumes that expenditures in the three general spend areas affect all clinical areas (healthy individuals, social care needs and other areas of spend, see Table 1). The inclusion of different combinations of general spend is determined in the SFA model. General spending is included if it enables the model to split error between statistical noise and inefficiency [24].

Some variables used in both models, notably mortality, are only available at the geographical unit called the Local Authority (LA). We mapped these data from the 326 district level LAs to the 151 PCTs, using a method based on Census 2011 population data. We tested this mapping method with population and deprivation data [29, 30], which are available at both LA and PCT level. Mapping LA to PCT populations produced estimates identical to available PCT data except for small differences in six PCTs located in one area (Birmingham). Mapping LA IMDs to PCTs produced means identical to those available for PCTs, albeit with a slightly smaller standard deviation.

Some of the non-mortality outcome variables are only available for Clinical Commissioning Groups (CCGs). CCGs replaced PCTs in 2013 and covered geographical areas different to both PCTs and LAs. We used a method published by the National Audit Office to map CCG-level outcomes [31], based on the 2012 population, to PCTs. We therefore have geographically consistent areas (at the PCT geographical unit level) for the QR and the DEA analyses.

## Results

### Quantile regression

**Fig. 2:**
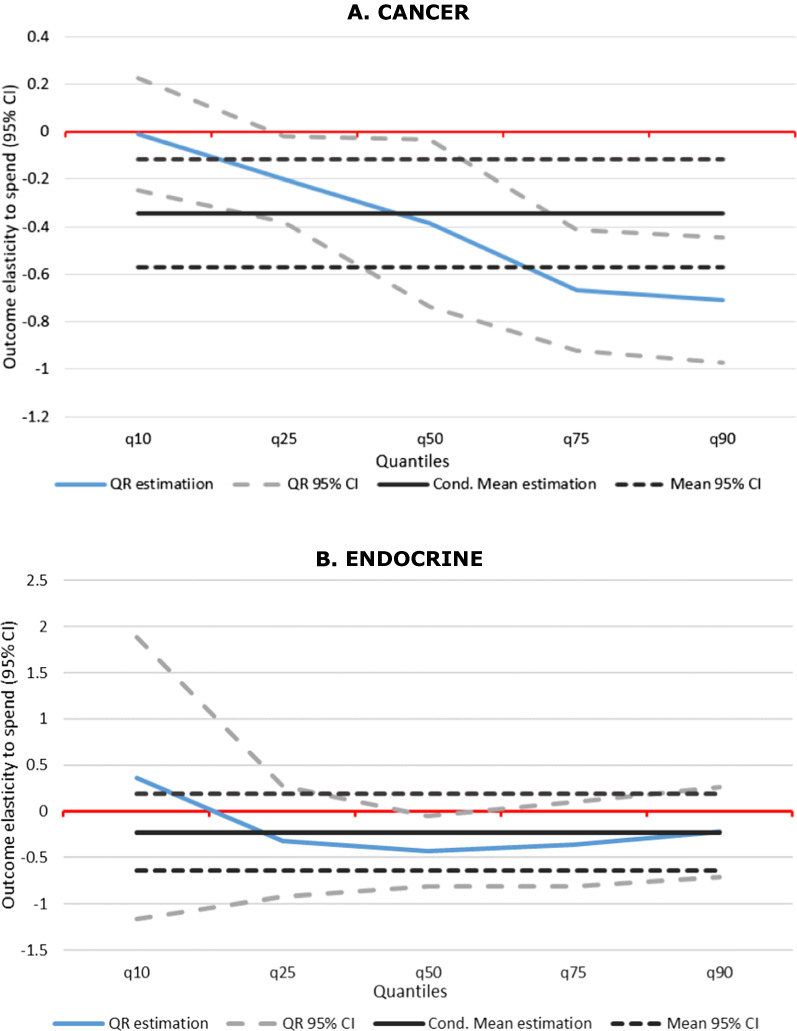
Quantile Regression Results for Cancer and Endocrine Diseases

The QR results indicate that expenditure/outcome relationships differ within both geographical areas (PCTs) and clinical areas (PBCs). The results of QR are presented in detail in six figures in the Additional file 1 Appendix. Two examples, Cancer and Endocrine diseases, are shown in Fig. 2. It shows the average effect and 95% confidence interval obtained from the linear regression model estimated by GMM, represented by the black horizontal lines. This is compared with the QR model estimated at five quantiles (0.1, 0.25, 0.5, 0.75, 0.9) represented by the blue line, also with 95% confidence intervals.

Detailed results for the model specification, estimation methods, and statistical tests (Endogeneity and Hansen overidentification tests) supporting the choice of IVs are presented in Additional file 2 Supplementary Material: Tables S2 to S7. These tables present Lomas et al. (2019) and our results for the same specification of the outcome equations. We make the final choice of IVs according to overidentification tests, and the selected IVs are specified at the bottom of Additional file 2 Supplementary Material: Tables S2 to S7 of the statistical validity of the IVs. These verify that they affect mortality only through changing the expenditure allocation and are therefore an exogenous source of variation in health expenditure per head, uncorrelated with potentially remaining unobservable factors determining mortality. Our choice of instruments may differ from Lomas et al. [4] as the authors do not disclose their choice.

The average return to spend in Cancer, reflected in the conditional mean mortality elasticity at 0.35, is only representative of the median of the QR distribution. For PCTs with low mortality rates, mortality elasticity is lower, that is less reduction in cancer mortality from increasing expenditure. In contrast, at the upper tail represented by the conditional effect on quantiles 0.75 and 0.90, elasticity is significantly larger: for those PCTs with the largest SYLLR the return to spend is about a 0.7% reduction in mortality for a 1% increase in spend per head, double the return of 0.35% at the mean and median.

For Endocrine a significant reduction of 0.43% in SYLLR for a 1% increase in spend per head is observed at the median. However, the effect of PBC spend per head on mortality is mostly stable along the mortality distribution. Additional file 1 Appendix: Figures A1 to A6 show that this is also the case for Respiratory and Gastrointestinal. Here, the average effect is a 1.5% decrease in SYLLR for a 1% increase in PBC spend per head, close to the QR estimates for the median. The effects are, however, more precisely estimated by QR in Circulatory which shows statistically significant larger reductions in mortality for PCTs with high rather than low mortality rates. For Infectious diseases however, expenditure increases have a higher effect on mortality for PCTs with low mortality rates. This may reflect the contagious nature of disease, such that preventive measures in low and mid risk populations are more effective in preventing mortality.

Our mean outcome elasticities from GMM unweighted estimates range from − 1.7 for PBCs gastrointestinal and respiratory to − 0.22 to PBC endocrine. These are similar to those obtained from GMM models using socioeconomic variables as instruments [4], and from methods based on the funding rule instruments [16]. The implied all-cause elasticities from [4] are also comparable with the directly estimated all-cause elasticities obtained using methods more robust to small-sample bias [15].

### Data envelopment analysis

The DEA results identify differences in efficiency across PCTs and within PCTs by PBCs. The three non-parametric tests consistently accept the less restrictive assumption of VRS indicating non-constant returns to scale.

PCTs are neither efficient nor inefficient over all of their activities. Most are efficient in some areas and less efficient in others. Of the 101 PCTs for which efficiency scores could be estimated for all seven PBCs, only two were fully efficient in every one (efficiency scores provided in Additional file 2 Supplementary Material: Table S8).

**Fig. 3:**
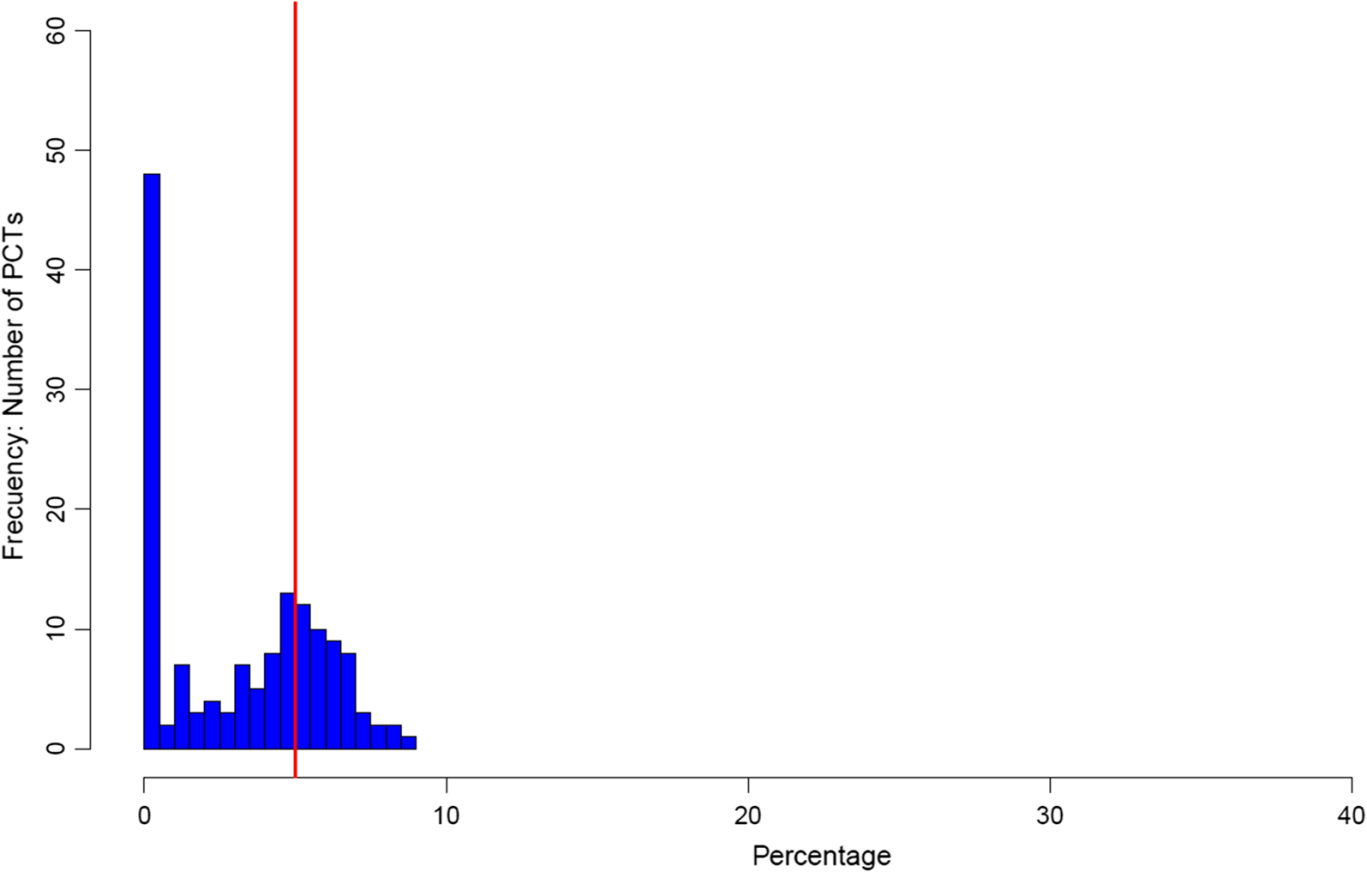
Percentage decrease in expenditure per year that could be possible without affecting health outcomes: Cancer. *(Adjusting for environmental variables)*

Figure 3 presents the DEA results for the case of Cancer. For the seven clinical areas, DEA results are illustrated through Figures A7 to A13 included in the Additional file 1 Appendix. Figure 3 shows the number of PCTs according to the proportional reduction in expenditure that the DEA scores indicate can be achieved without affecting outcomes (∆*/Ω** in Fig. 1), expressed as a percentage. On the left is the number of fully efficient PCTs, with increasingly less efficient PCTs extending to the right. The red line is a 5% reduction in expenditure without affecting outcomes. The more PCTs to the right of this, the greater the inefficiency in that PBC.

The differences between PBCs in the pattern of efficiency across PCTs are shown in Additional file 2 Supplementary Material: Table S9. This displays and ranks the PBC according to (1) the percentage of fully efficient PCTs and (2) the percentage of PCTs in that PBC that have scope to improve efficiency, that is they can decrease expenditure by more than 5% without affecting health outcomes. These rankings do not always match. For example, Maternity has more efficient PCTs than Gastrointestinal. However, Gastrointestinal has less scope for efficiency improvement; a much smaller percentage of PCTs would be able to decrease expenditure by 5% or more without affecting outcomes.

### Comparison of DEA and QR

For the five PBCs also analysed in DEA, the absolute value of QR mortality elasticities is positively correlated with the mortality level. There is also a systematic negative correlation between the elasticities and PCTs’ DEA efficiency scores (Additional file 2 Supplementary Material: Table S10), which are statistically significant except for Endocrine.

The last three columns of Additional file 2 Supplementary Material: Table S10 compare the mean mortality elasticities in efficient and inefficient PCTs. Efficient PCTs have a lower absolute mortality elasticity, which is consistent with the ranking correlations. These mean-comparison t-tests are again all significant apart from Endocrine. An increase in spend in each PBC results in a lower reduction in mortality in efficient PCTs than in others. This indicates that PCTs operating efficiently in a PBC have lower rates of mortality. For most PBCs, the lower the mortality, the lower the mortality elasticity, implying that it is harder to achieve additional reductions in mortality in PCTs that are already operating efficiently.

## Discussion, policy implications, and limitations

The results have implications both for our understanding of health production and for estimation and use of a single health-care system wide cost-effectiveness threshold.

The QR results suggest that the effect of increasing health expenditure per head on the mortality rate, as measured by the outcome elasticity, differs between PBCs (clinical areas) and between PCTs (geographical units) within PBCs. The DEA results indicate differences in efficiency across PCTs and within PCTs by PBC indicating that PCTs have differing abilities to achieve best practice performance. We found a negative correlation between mortality elasticity measured by QR and efficiency measured by DEA: if all PBCs increased their spending by the same percentage, the percentage reduction in mortality would be *lower* in efficient PCTs than in others. A plausible explanation is that PCTs operating efficiently in a PBC have lower rates of mortality and, for most disease areas, the lower the mortality, the harder it is to achieve additional reductions. We can note that Edney et al*.* [13] in their QR analysis found that marginal returns on Australian public health spending were significantly greater for areas with poorer health outcomes compared to areas with better health outcomes. More generally, the results indicate there is not one aggregate health production function applicable to the whole health care system but many such functions, varying by clinical area and geographical unit.

This means that opportunity costs vary, although the way in which they vary is complex. The negative relationship between efficiency, mortality rates and outcome elasticities implies that less efficient PCTs have greater opportunities to improve outcomes for a given percentage change in expenditure. More efficient PCTs tend to have lower rates of mortality and find it harder to achieve further reductions and so have a lower potential to achieve improved outcomes from higher expenditure than their inefficient counterparts, when focusing on reducing mortality. For less efficient PCTs, the expenditure required to fund a new technology might be provided, at least in part, by improving efficiency without reductions in outcomes, suggesting a lower opportunity cost in terms of reduced health. But if less efficient PCTs do not respond this way, an effective reduction in expenditure may have a greater effect on outcomes (increased mortality) in these PCTs than implied by the average, that is more health is given up.

The main purpose of recent literature estimating outcome elasticities has been to derive a system wide cost-effectiveness threshold, by identifying a health production function which shows the marginal productivity of current health expenditure. This requires the *mean* relationship over the range of identified observations to be interpreted as a *marginal* relationship. Methods commonly used to model the conditional expectation can only estimate a single elasticity, and no data transformation, such as the logarithmic, overcomes the problem that a constant elasticity must be assumed to reflect a true marginal response. Our QR results cast doubt on this key assumption.

From a practical point of view, it would clearly be advantageous to identify and use a single system-wide threshold. The QR results suggest another route into estimating such a threshold, weighting by the absolute levels of mortality in clinical areas. However, it is unclear how this would differ from the average linear regression estimate, which is close to the median QR estimate. It depends on the attributes of the underlying production functions in each clinical area, including assumptions about how efficiency varies within and between the commissioning units, both of which will impact on the QR results.

The QR approach also enables us to incorporate inequality into an assessment of the impact of a technology. The variability of mortality elasticity and coefficients representing local health needs and deprivation indicators at different quantiles can be interpreted as an inequality “gradient” supporting analysis using distributional cost-effectiveness analysis [32–34].

However, an inescapable conclusion is that efficiency and equity in allocation of health care resources would be best served by having different cost-effectiveness thresholds for different disease groups and different geographical areas. This may appear to conflict with the desire to ensure that people have the same access to services provided by a health system given their health need, wherever they live. But if opportunity costs do differ between different disease groups and geographical areas, a common threshold will not serve that equity aim. Using that threshold to make sure some services are uniformly available risks increasing the disparity of availability of the remaining services.

It is unclear how a health care system would incorporate multiple cost-effectiveness thresholds into decision making about new or existing technologies. However, adopting a single threshold does not make the problem of variations in health system opportunity cost disappear.

Our analysis has applications beyond consideration of the implications for system wide estimates of the threshold. The DEA results indicate the potential value of using this approach in local commissioning to identify clinical areas where local provision appears not to be efficient when compared with other geographical units, and potential improvements in efficiency may be realisable. The QR results indicate how local thresholds could be estimated to inform decision making by local commissioners within their budgets, as only a relatively small component is taken up by the use of nationally mandated technologies.

There are limitations to our study. Firstly, the scope is limited to analysis of outcome elasticities, which represent the relative effect of expenditure on health. Although we have not translated these relative effects to the absolute changes in health gain (changes in QALYs) for a given change in budget, the implications for the measurement of opportunity cost are clear.

Secondly, the econometric models, both the DEA and the QR, apply accepted methods, using validated IVs for health expenditure, to control for the confounding effects of other health need and socioeconomic variables, and for the endogeneity bias of the outcome elasticities. We acknowledge the limitations of these methods and have commented on the robustness of our results as compared to those using different methods applied to the same data. When directly comparable, our results are very similar to those in studies we have referenced that use other methods which consider additional statistical problems, for example small sample bias and instrument redundancy.

Thirdly, comparing the DEA and QR is challenging given they are very different measures. Our comparison is a non-parametric relationship of PCT ranking according to these measures, and a simple comparison of mean outcome elasticities between fully efficient and non-efficient PCTs. The results are consistent.

Fourthly, and arguably, the main limitation of our study, common to all studies using expenditure and mortality across health locations, results from the limitations of, and quality of, the data available on inputs and on outcomes. For example, we have used data up to 2012/13 because NHS reorganisation centralised purchasing for a number of hospital services from 2013, and expenditure for these services is no longer broken down by geographical and clinical area.

## Conclusions

Considering the opportunity cost of new technologies should be an essential element of decision making about their use. Estimates of such opportunity costs have been published using regression models of expenditures and mortality that may not be correctly specified and do not account for possible inefficiencies in health production.

Our estimates capture the clinical and geographical variability of opportunity costs from variations in health production functions. They also show that health care administrative units with lower mortality and higher efficiency have lower outcome elasticities, suggesting diminishing returns from health expenditure in reducing mortality.

Our results caution against relying on evidence which assumes a single system-wide health production function. One interpretation is that there should be different cost-effectiveness thresholds for different disease areas and different geographical areas, although incorporating multiple cost-effectiveness thresholds into a health system could be complicated.

In most health care systems, many decisions about provision are, however, not made centrally. Using DEA and QR analysis to understand the variability in opportunity cost will help policy makers target efficiency improvements and set realistic targets for local and clinical area health improvements from increased expenditure.

## Supplementary Information

**Additional file 1.** Appendix: Figures of Quantile regression and DEA Results.

**Additional file 2. **Supplementary Material.

## Data Availability

The datasets supporting the conclusions of this article are included within the article and its additional files. Source data is from public domain sources which are referenced.
