# Supplementary material for: Estimating health system opportunity costs: the role of non-linearities and inefficiency
Source: Cost Eff Resour Alloc. 2022 Oct 29;20:56. doi: 10.1186/s12962-022-00391-y (PMC9617442; doi:10.1186/s12962-022-00391-y)

**Appendix**

**Figures A1-A6. Quantile Regression**

**Figure A1. Infectious**


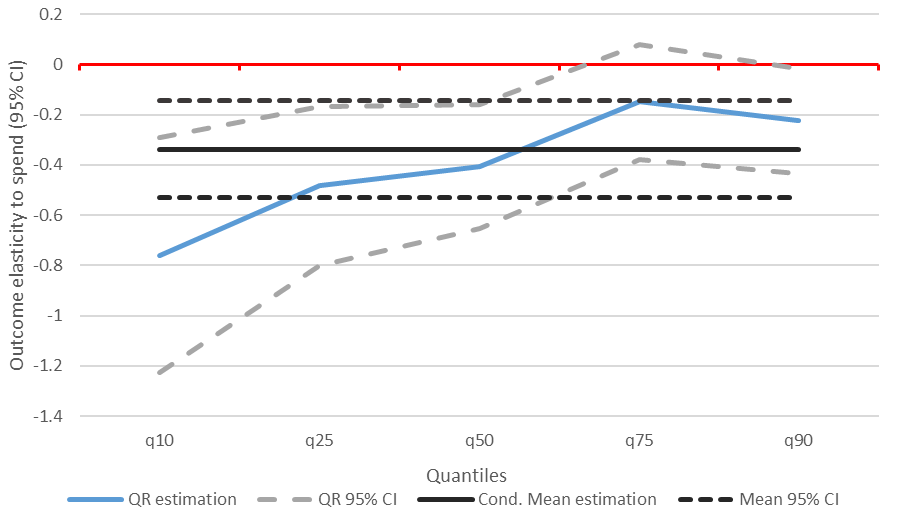


**Figure A3. Cancer**


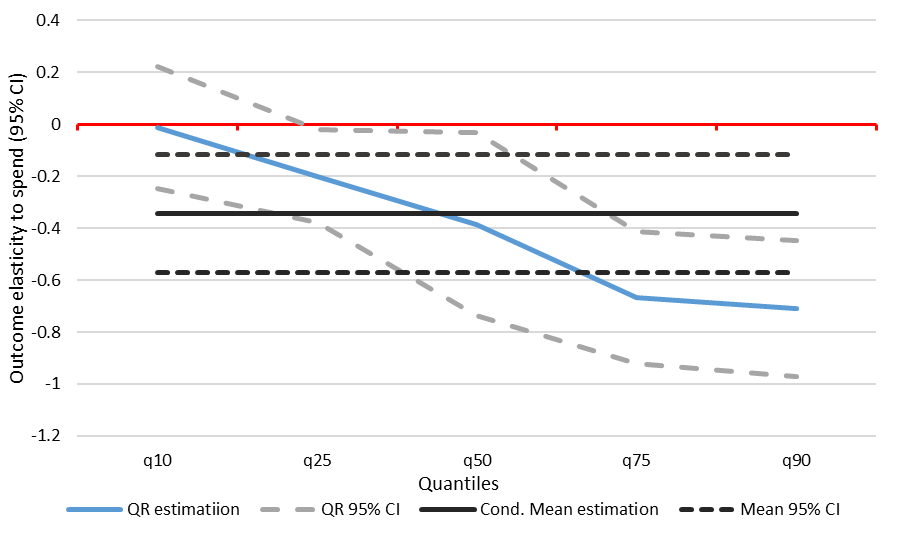


**Figure A5. Endocrine**


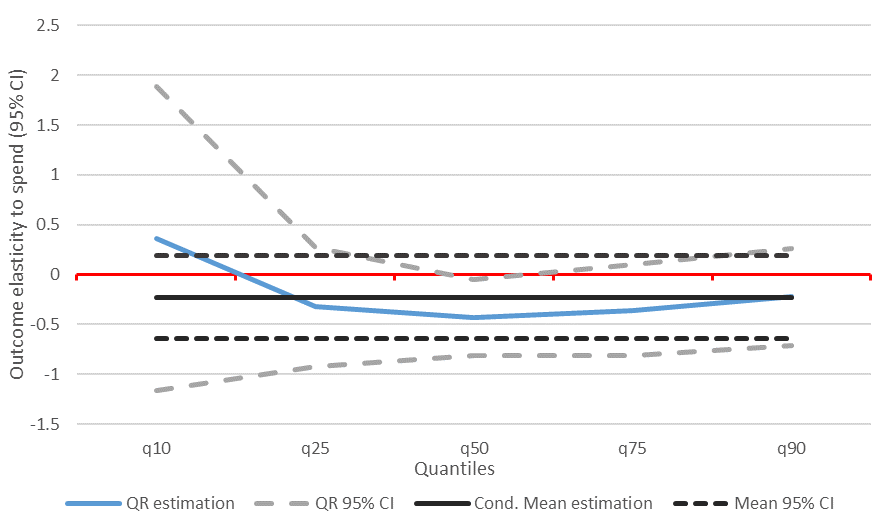


**Figure A2. Circulatory**


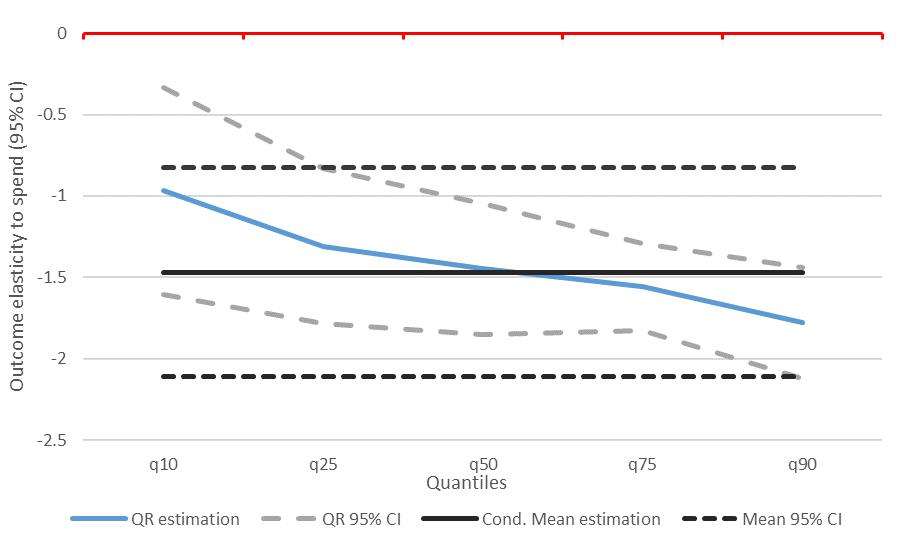


**Figure A4. Respiratory**
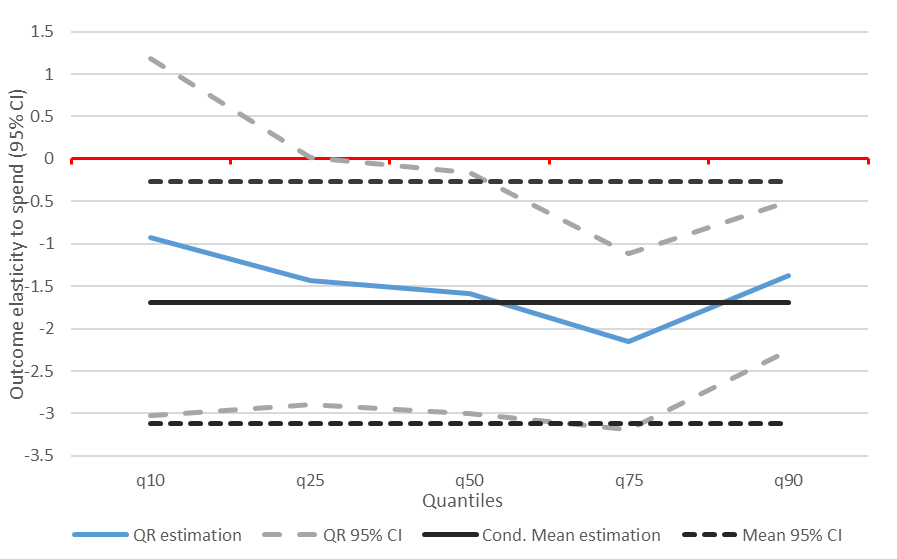


**Figure A6. Gastrointestinal**
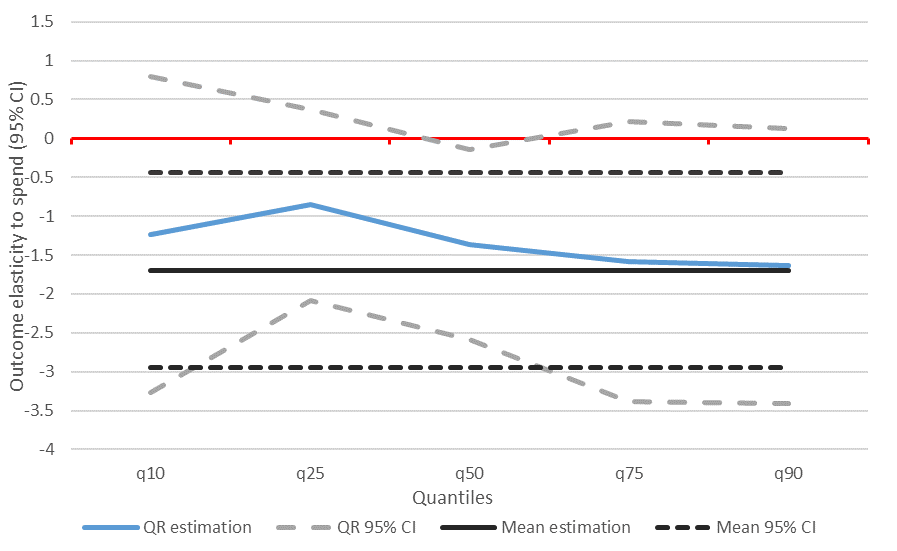


**Figures A7-A13. DEA results (with Environmental Variables)**

Percentage decrease in expenditure that could be possible without affecting health outcomes. Inefficiency level indicates percentage of PCTs which can decrease per-capita expenditures per year in at least 5% without affecting health outcomes correspond to all observations located on the right of the red line.

Figure A7. Mental Health

2 missing in variable MH_Employment (5F7 and 5QT)

5 outliers (PCTs 5A3, 5HQ, 5NW, 5PY and 5QC).

Extent_2010 included as environmental variable instead of IMD_2010

Efficiency: 52 (36.1%) efficient PCTs out of 144.

Inefficiency: 48 (33.3%)


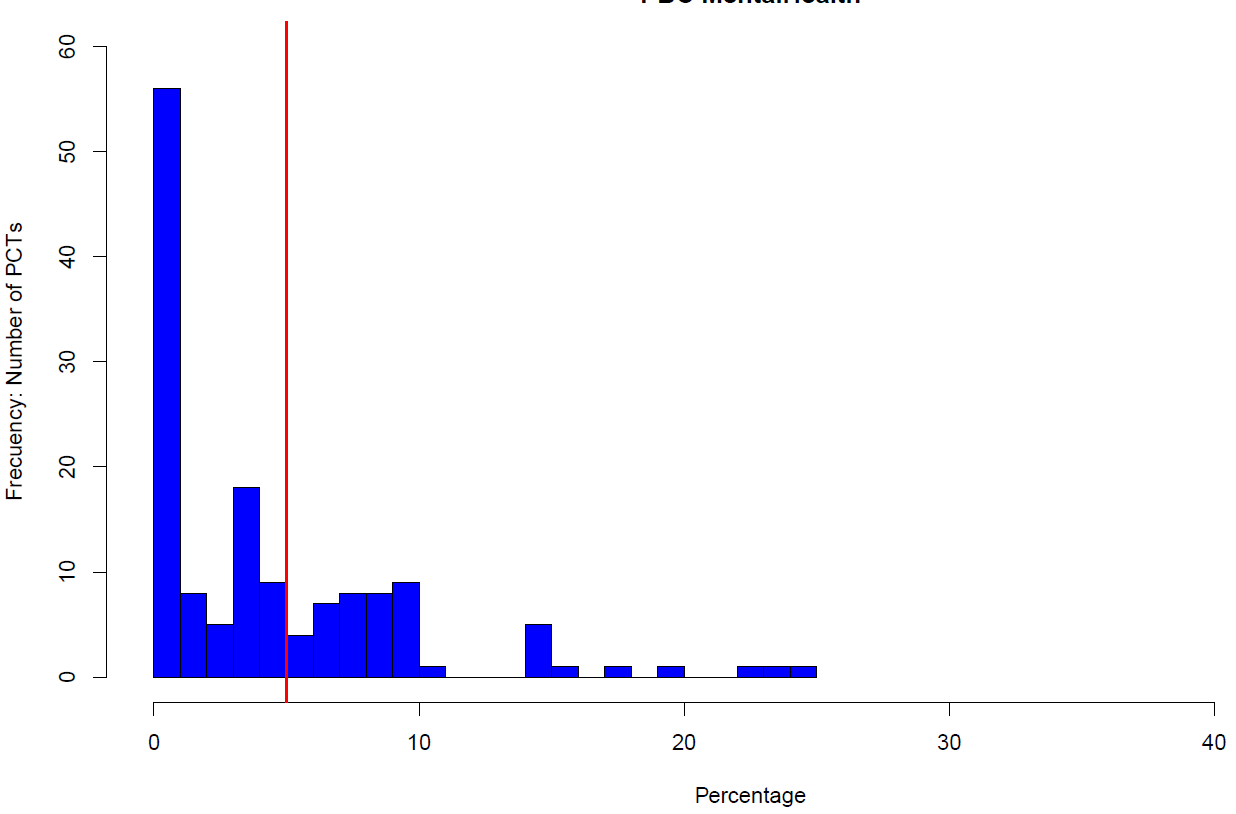


Figure A9. Maternity

1 missing in the variable NeonatalMort_2014_INV (5D7).

4 outliers (5F7, 5M2, 5NW and 5QC).

Efficiency: 38 (26%) efficient PCTs out of 146.

Inefficiency: 89 (60.9%)


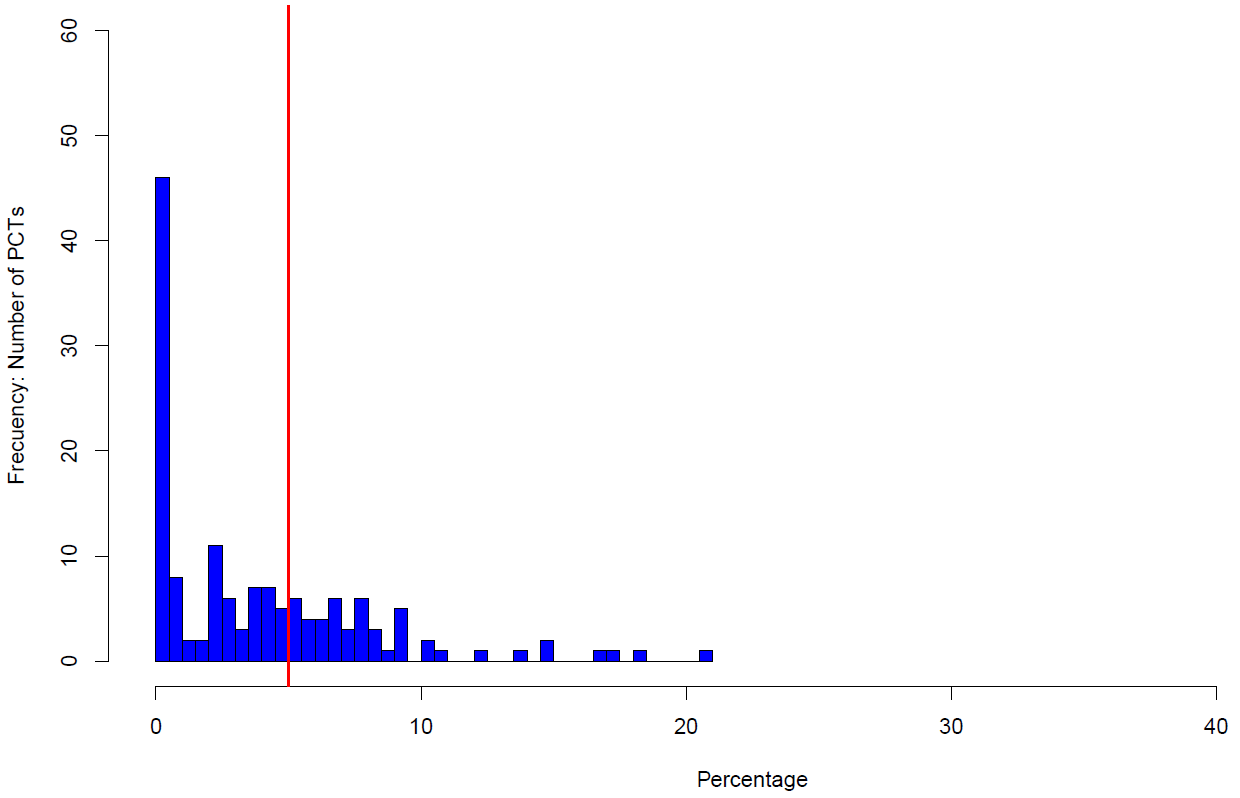


Figure A8. Cancer

No missing; 4 outliers (outliers (5HY, 5LE, 5NA and 5PQ).

Efficiency: 44 (29.9%) efficient PCTs out of 147.

Inefficiency: 47 (31.9%)


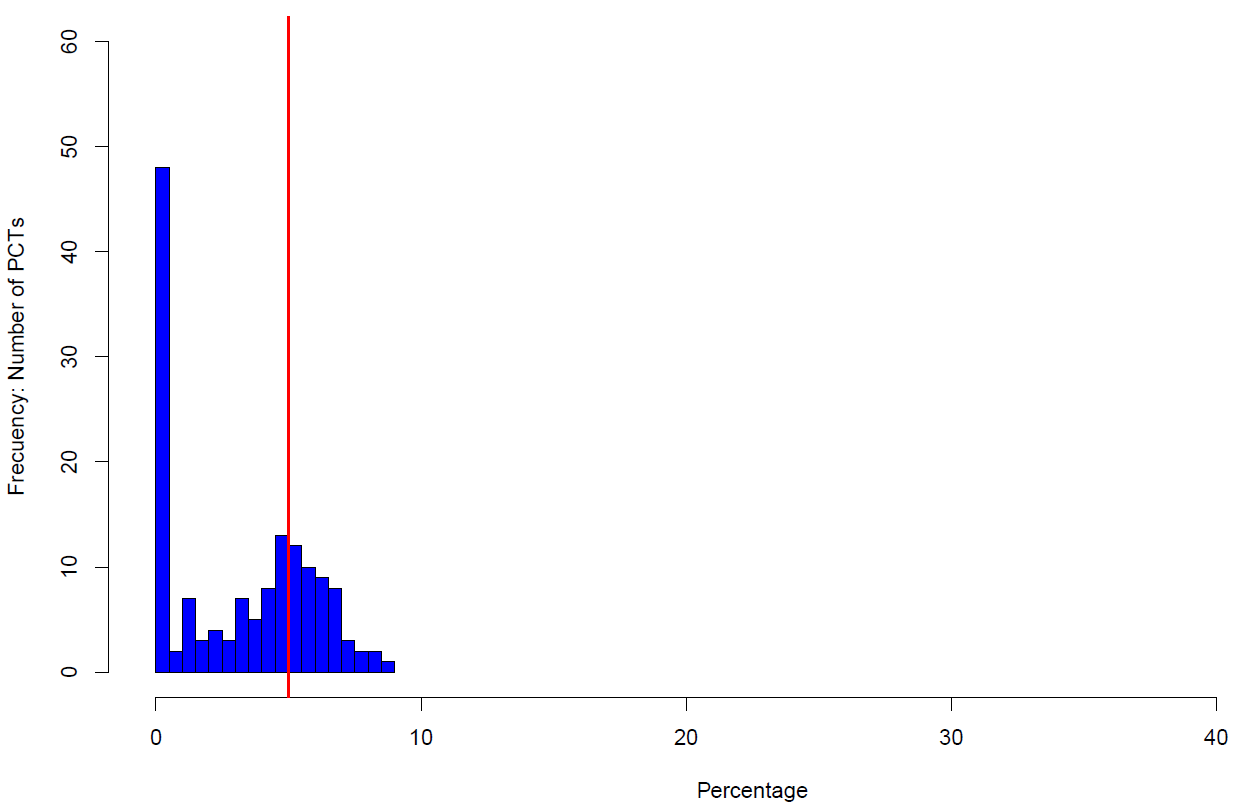


Figure A10. Gastrointestinal

No missing; 4 outliers (5C3, 5MX, 5PL and 5QF).

Efficiency: 39 (26.5 %) efficient PCTs out of 147.

Inefficiency: 10 (6.8 %)


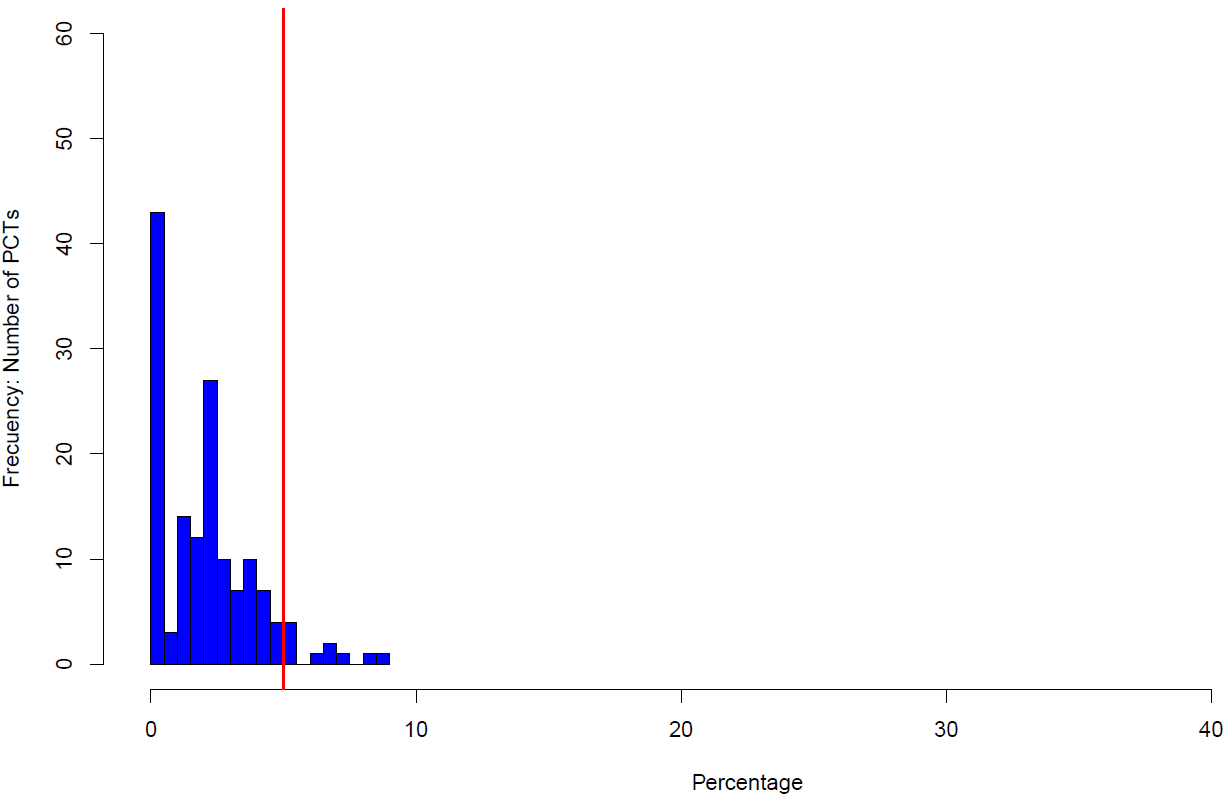


**Figure A11. Cardiovascular**

23 missing in in CardiacRehab_2014 and 1 missing in Stroke_discharge_2014.

The environmental variable TargetDistance_2010 was excluded

7 outliers: Seven outliers (5EM, 5J6, 5LD, 5LE, 5N7, 5PX and 5QF). Efficiency: 52 (43.2%) efficient PCTs out of 120.

Inefficiency: 43 (35.8%)


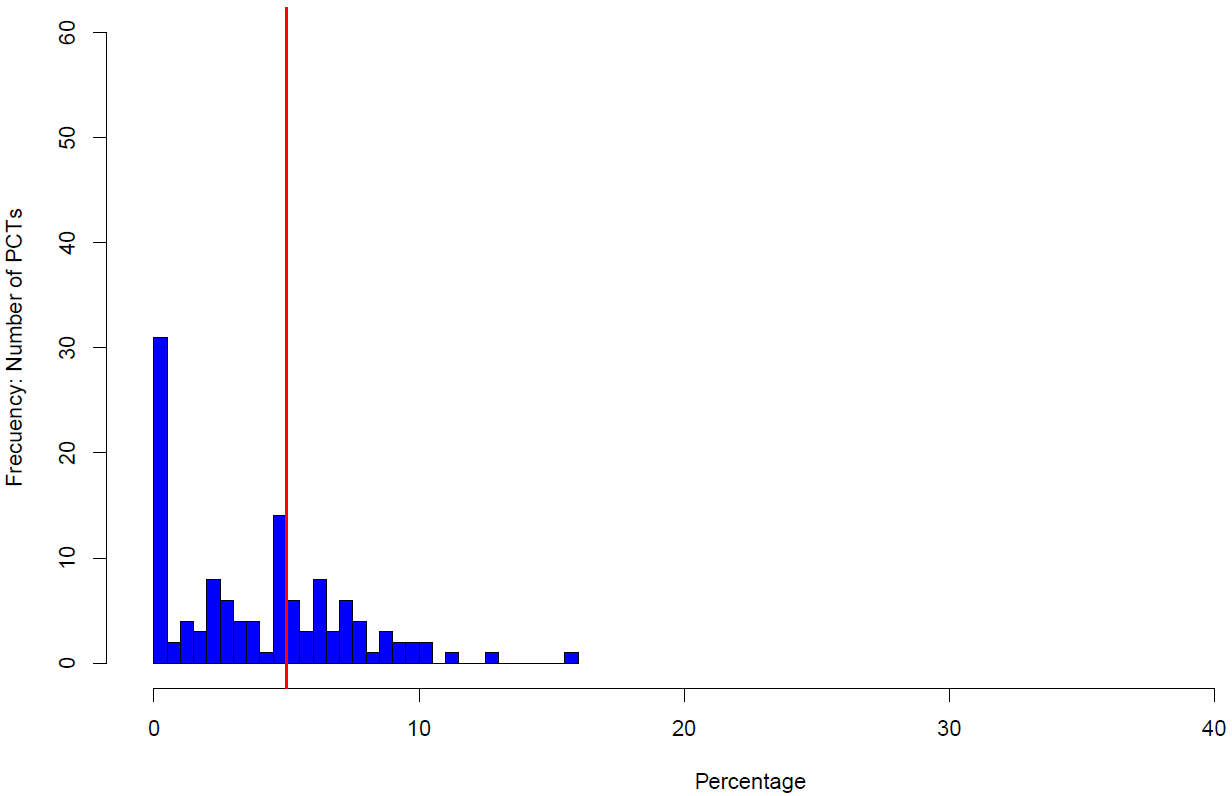


**Figure A13. Respiratory**

1 missing in EmergencyRespiratoryChild_2014_INV

4 outliers (5C3, 5C9, 5N7 and 5QP)

Extent_2010 included as environmental variable instead of IMD_2010

Efficiency: 48 (32.9%) efficient PCTs out of 146.

Inefficiency: 34 (23.3%)


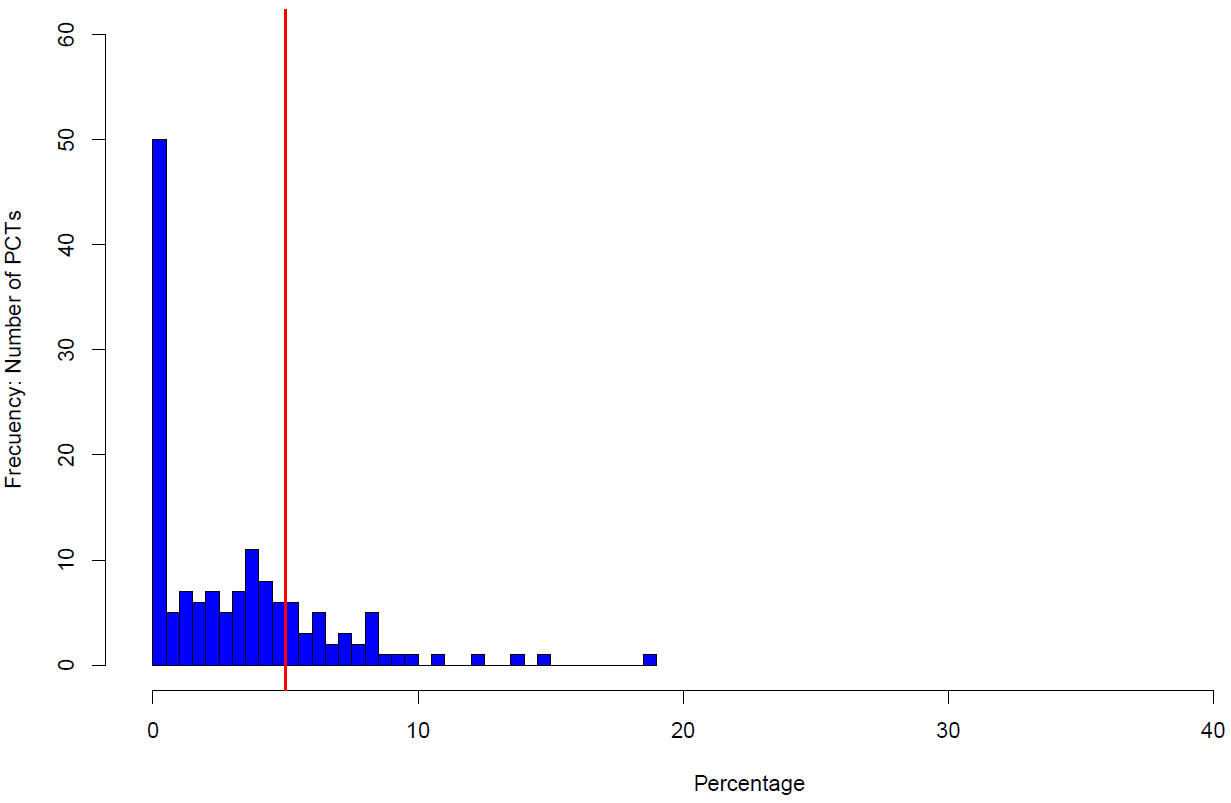


Figure A12. Endocrine

1 missing in DiabComplications_2014_INV

5 outliers (5D9, 5F7, 5H8, 5L1 and 5QQ).

Efficiency: 34 (23.3%) efficient PCTs out of 146.

Inefficiency: 24 (16.44%)

.

.


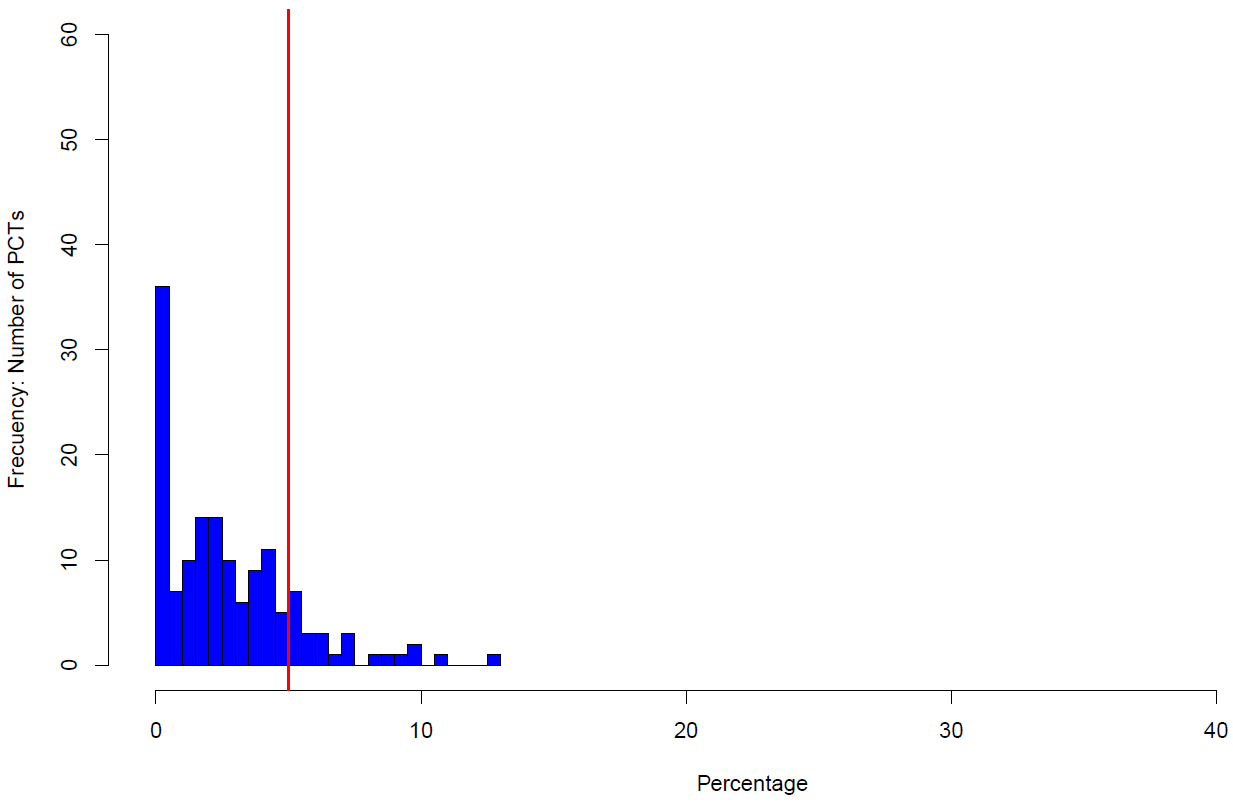

Supplement: Supplementary file 1 — Additional file 1. Appendix: Figures of Quantile regression and DEA Results. [file 12962_2022_391_MOESM1_ESM.docx]
