# Supplementary material for: Estimating health system opportunity costs: the role of non-linearities and inefficiency
Source: Cost Eff Resour Alloc. 2022 Oct 29;20:56. doi: 10.1186/s12962-022-00391-y (PMC9617442; doi:10.1186/s12962-022-00391-y)
Supplement: Supplementary file 2 — Additional file 2. Supplementary Material. [file 12962_2022_391_MOESM2_ESM.docx]

**Supplementary Material**

**Can non-linearities and inefficiency be accounted for when estimating health system opportunity costs?**

[1. Programme Budget Categories 1](#_Toc110689381)

[2. Quantile Regressions 3](#_Toc110689382)

[3. Health outcomes considered in DEA models 10](#_Toc110689383)

[4. Methodology for the DEA: three-step procedure 13](#_Toc110689384)

[4.1. First Step: Initial DEA 13](#_Toc110689385)

[4.2. Second Step: Stochastic frontier analysis (SFA) 13](#_Toc110689386)

[4.3. Third Step: Adjusted DEA 15](#_Toc110689387)

[5. Efficiency Scores 16](#_Toc110689388)

[6. Efficiency rankings 19](#_Toc110689389)

[7. Comparison of DEA and QR estimations 19](#_Toc110689390)

# Programme Budget Categories

The Department of Health and Social Care in England established national Programme Budgeting in order to provide information on the use of health care resources by disease area. Primary Care Trusts (PCTs) provided a breakdown of their expenditure allocated to specific healthcare conditions. These conditions were grouped into Programme Budget Categories (PBCs) based on the World Health Organisation International Classification of Diseases. The groupings making up the individual PBCs are set out in Table S1.

Table S1. Programme Budget Categories (PBCs)

| **PBC CODE** | **PBC NAME** | **PBC CODE** | **PBC NAME** |
| --- | --- | --- | --- |
| **PBC 1** | **Infectious diseases** | **PBC 11** | **Problems of the respiratory system** |
| 01a | HIV and AIDS | 11a | Obstructive airways disease |
| 01x | Infectious diseases (Other) | 11b | Asthma |
| **PBC 2** | **Cancers and tumours** | 11x | Problems of the respiratory system (other) |
| 02a | Head or neck cancers | **PBC 12** | **Dental problems** |
| 02b | Upper gastro intestinal cancers | **PBC 13** | **Problems of the gastro intestinal system** |
| 02c | Lower gastro intestinal cancers | 13a | Upper gastro intestinal system problems |
| 02d | Lung cancers | 13b | Lower gastro intestinal system problems |
| 02e | Skin cancers | 13c | Hepatobiliary problems |
| 02f | Breast cancers | 13x | Problems of the gastro intestinal system (other) |
| 02g | Gynaecological cancers | **PBC 14** | **Problems of the skin** |
| 02h | Urological cancers | 14a | Burns |
| 02i | Haematological cancers | 14x | Problems of the skin (other) |
| 02x | Cancers and tumours (other) | **PBC 15** | **Problems of the musculoskeletal system** |
| **PBC 3** | **Disorders of blood** | **PBC 16** | **Problems due to trauma and injuries** |
| **PBC 4** | **Endocrine, nutritional and metabolic problems** | **PBC 17** | **Problems of the genito urinary system** |
| 04a | Diabetes | 17a | Genital tract problems |
| 04b | Endocrine | 17b | Renal problems |
| 04x | Endocrine, nutritional and metabolic problems (other) | 17c | Sexually transmitted infections |
| **PBC 5** | **Mental health disorders** | 17x | Problems of genito urinary system (other) |
| 05a | Substance misuse | **PBC 18** | **Maternity and reproductive health** |
| 05b | Organic mental disorders | **PBC 19** | **Conditions of neonates** |
| 05c | Psychotic disorders | **PBC 20** | **Adverse effects and poisoning** |
| 05d | Child and adolescent mental health disorders | 20a | Unintended consequences of treatment |
| 05x | Mental health disorders (other) | 20b | Poisoning |
| **PBC 6** | **Problems of learning disability** | 20c | Violence |
| **PBC 7** | **Neurological** | 20x | Adverse effects and poisoning (other) |
| 07a | Chronic pain | **PBC 21** | **Healthy individuals** |
| 07x | Neurological (other) | **PBC 22** | **Social care needs** |
| **PBC 8** | **Problems of vision** | **PBC 23** | **Other** |
| **PBC 9** | **Problems of hearing** | 23a | GMS/PMS |
| **PBC 10** | **Problems of circulation** | 23x | Miscellaneous |
| 10a | Coronary heart disease |  |  |
| 10b | Cerebrovascular disease |  |  |
| 10c | Problems of rhythm |  |  |
| 10x | Problems of circulation (other) |  |  |

Source: NHS England, 2018. Programme Budgeting. Accessed 27/06/2018. https://www.england.nhs.uk/resources/resources-for-ccgs/prog-budgeting/

# Quantile Regressions

The specification of the model estimated on the QR are taken from the preferred outcome specification of Lomas et al. [4] for the 6 selected PBCs.

The dependent variable is the three-year average SYLLR as presented by ONS Compendium Statistics for years 2012-2013-2014, These same data are used by Claxton et al. [35] to define their dependent variable, the only difference is the mapping used and the final geographical area chosen to represent mortality outcomes. They use original data at top-tier local authority (LA) for 152 LAs (unitary authority, metropolitan district, London borough, counties). They also use these mortality data mapped to PCT-level area. We use original data for 326 LAs (Local authority districts, unitary authority, metropolitan district, London borough) and map these to PCT-level according to the mapping method described based on 2011 Census population.

Regarding the explanatory variables, we use for each of the 6 PBCs the same explanatory variables as used by Lomas et al. [4], except some differences in the measurement year for CARAN and HIV needs. These explanatory variables and their descriptive statistics have been presented in Table 5.

The final instruments used by Lomas et al. [4] are not available, so that we have chosen several specifications from instruments presented in Table 4 (deprivation and socioeconomic variables) to achieve overidentification and meeting this overidentification test which indicates validity of the instruments. These final instruments used are detailed in results presented in this Supplementary Material.

The following tables detail the estimations presented in Figures A1 to A6 in the Appendix to the main paper. Results published by Lomas et al. [4] are presented in the first column for the sake of comparison. Estimates of the conditional mean model are presented unweighted to use as benchmark of comparison with QR estimates, since QR estimation does not allow weights. Mean estimates are also weighted to consider different PCT size as Lomas et al. [4] do. Most of the explanatory variables in out model coincide with those selected in York team’ preferred specification model, although our variables on health needs (HIV need and CARAN need refer to year 2011/12 instead of 2012/13 in the Claxton et al. [35] estimations. Figures A1 to A6 represent a horizontal line for the conditional mean estimate (unweighted) of the outcome elasticity to PBC spend (coefficient of variable lg`PBC’_1213netpoppheadOHP) in each one of the six PBCs. The blue line links the QR estimates of this outcome elasticity in each table. Figures also show the 95% CI corresponding to the estimated standard deviation (in brackets).

**Table S2**

| **Lomas et al. [4], Claxton et al. [35]** | |  | **ESTIMATES** | | | | | | | |
| --- | --- | --- | --- | --- | --- | --- | --- | --- | --- | --- |
| **PBC 1 Infectious** | |  | **PBC 1 Infectious** | | | | | | | |
| 2012/13 spend | |  | 2012/13 spend | | | | | | | |
| SYLLR 2012/13/14 | |  | SYLL 2012/13/14 | | | | | | | |
| Weighted | |  |  | unweighted | weighted |  |  |  |  |  |
| OLS mean | |  |  | OLS mean | | Quantile Regression | | | | |
| LA- level | |  | PCT-level |  |  |  |  |  |  |  |
|  | mean | |  | mean | mean | q50 | q10 | q25 | q75 | q90 |
| lLAg1_1213netpoppheadOHP | -0.362*** | | lg1_1213netpoppheadOHP | -0.3379*** | -0.3218*** | -0.4083** | -0.7593** | -0.4844** | -0.1497 | -0.2248* |
|  | [0.089] | |  | [0.0981] | [0.0861] | [0.1249] | [0.2365] | [0.1598] | [0.1165] | [0.1061] |
| lLAHIVneedph | 0.276*** | | lHIVneedprev | 0.6851*** | 0.6667*** | 0.6809*** | 1.1703*** | 0.8145*** | 0.4355** | 0.4858*** |
|  | [0.045] | |  | [0.1107] | [0.1036] | [0.1159] | [0.2256] | [0.1839] | [0.1310] | [0.1100] |
| lIMD2010 | 0.649*** | | lIMD2010 | 0.4513*** | 0.4616*** | 0.4545*** | 0.3343*** | 0.3659** | 0.5455*** | 0.6310*** |
|  | [0.064] | |  | [0.0620] | [0.0526] | [0.0607] | [0.0886] | [0.1202] | [0.0682] | [0.0799] |
| lLONEPENH | -0.177 | | lLONE65andover | -0.0182 | -0.0262 | -0.2368 | 0.3559 | -0.253 | -0.0235 | 0.0615 |
|  | [0.183] | |  | [0.1847] | [0.1775] | [0.1923] | [0.2193] | [0.3960] | [0.1618] | [0.1250] |
| _cons | 0.698 | | _cons | 1.4885** | 1.3989** | 1.2447* | 3.7004*** | 1.6047 | 0.7468 | 1.0102* |
|  | [0.437] | |  | [0.5385] | [0.5065] | [0.5008] | [0.9784] | [1.0515] | [0.4750] | [0.4959] |
|  |  | |  |  |  |  |  |  |  |  |
| N. Observations | 147 | | N. Observations | 151 | 151 | 151 | 151 | 151 | 151 | 151 |
| R-squared | 0.582 | | R-squared/Pseudo R-squared | 0.587 | 0.612 | 0.394 | 0.291 | 0.333 | 0.427 | 0.406 |
|  | |  | Test H0: spend coef=q50 | | |  |  |  | * |  |
|  | |  | Test H0 spend coef=q10 | |  |  |  |  | * | * |
|  | |  | Test H0 spend coef=q25 | |  |  |  |  | * |  |
|  | |  | Test H0 spend coef=q75 | |  |  |  |  |  |  |

Notes:

Significance levels: * for p<.05, ** for p<.01, and *** for p<.001.

**Table S3**

| **Lomas et al. [4], Claxton et al. [35]** | | **ESTIMATES**  **PBC 2 Cancer**  2012/13 spend  SYLLR 2012/13/14  instrument spend | | | | | | | | |
| --- | --- | --- | --- | --- | --- | --- | --- | --- | --- | --- |
| **PBC 2 Cancer** |  |  |  |  |  |  |  |  |  |  |
| 2012/13 spend | |  |  |  |  |  |  |  |  |  |
| SYLLR 2012/13/14 | |  |  |  |  |  |  |  |  |  |
| instrument spend | |  |  |  |  |  |  |  |  |  |
| weighted |  |  | | unweighted | weighted |  |  |  |  |  |
| IV second stage (GMM) | |  | IV second stage (GMM) | | | Quantile Regression | | | | |
| LA-level |  | PCT-level | |  |  |  |  |  |  |  |
|  | mean |  | | mean | mean | q50 | q10 | q25 | q75 | q90 |
| lLAg2_1213pheadOHP | -0.361** | lg2_1213netpoppheadOHP | | -0.3447** | -0.4693* | -0.3845* | -0.0107 | -0.1999* | -0.6669*** | -0.7091*** |
|  | [0.149] |  | | [0.1160] | [0.2076] | [0.1782] | [0.1189] | [0.0910] | [0.1288] | [0.1327] |
| lLACARANneed1213 | 1.023*** | lCARANneed | | 0.6779*** | 0.6313*** | 0.6818*** | 0.7722*** | 0.6882*** | 0.6370*** | 0.6421*** |
|  | [0.134] |  | | [0.0610] | [0.0920] | [0.0754] | [0.1203] | [0.0815] | [0.0460] | [0.0476] |
| _cons | 6.744*** | _cons | | 6.6738*** | 7.2532*** | 6.8596*** | 5.0199*** | 5.9565*** | 8.2265*** | 8.4428*** |
|  | [0.691] |  | | [0.5408] | [0.9640] | [0.8374] | [0.5541] | [0.4306] | [0.6002] | [0.6251] |
|  |  |  | |  |  |  |  |  |  |  |
| N. Observations | 149 | N. Observations | | 151 | 151 | 151 | 151 | 151 | 151 | 151 |
| R-squared |  | Pseudo R-squared | | |  | 0.44 | 0.38 | 0.39 | 0.49 | 0.51 |
| Endogeneity test | 8.48 | Endogeneity test statistic | | 8.26 | 16.22 |  |  |  |  |  |
| Endogeneity p-value | 0.004 | Endogeneity p-value | | 0.00 | 0.00 |  |  |  |  |  |
|  |  | Hansen-Sargan test | | 3.23 | 0.51 |  |  |  |  |  |
|  |  | Hansen-Sargan p-value | | 0.07 | 0.47 |  |  |  |  |  |
|  |  | Test H0: spend coef=q50 | | |  |  | * |  |  |  |
|  |  | Test H0 spend coef=q10 | | |  |  |  |  | *** | *** |
|  |  | Test H0 spend coef=q25 | | |  |  |  |  | *** | *** |
|  |  | Test H0 spend coef=q75 | | |  |  |  |  |  |  |

Notes:

Significance levels: * for p<.05, ** for p<.01, and *** for p<.001.

Instruments: lIMD2010, lLONEPARH

**Table S4**

| **Lomas et al. [4], Claxton et al. [35]** | | **ESTIMATES** | | | | | | | |
| --- | --- | --- | --- | --- | --- | --- | --- | --- | --- |
| **PBC 4 Endocrine** | | **PBC 4 Endocrine** | | | | | | | |
| 2012/13 spend | | 2012/13 spend | | | | | | | |
| SYLLR 2012/13/14 | | SYLLR 2012/13/14 | | | | | | | |
| instrument spend | |  |  |  |  |  |  |  |  |
| weighted |  |  | unweighted | weighted |  |  |  |  |  |
| IV second stage (GMM) | |  | OLS | | Quantile Regression | | | | |
| LA-level |  | PCT-level |  |  |  |  |  |  |  |
|  | mean |  | mean | mean | q50 | q10 | q25 | q75 | q90 |
| lLAg4_1213pheadOHP | -0.499 | lg4_1213netpoppheadOHP | -0.2284 | -0.2898 | -0.4328* | 0.3593 | -0.3253 | -0.3562 | -0.2218 |
|  | [0.349] |  | [0.2103] | [0.1686] | [0.1920] | [0.7700] | [0.3003] | [0.2326] | [0.2459] |
| lLAIMD2010 | 0.579*** | lIMD2010 | 0.5157*** | 0.4493*** | 0.5935*** | 0.6990* | 0.4058** | 0.4111*** | 0.4565*** |
|  | [0.116] |  | [0.0936] | [0.0896] | [0.0974] | [0.2951] | [0.1484] | [0.0669] | [0.1339] |
| LPROFOCCU | -0.409** | lPROFOCCU | -0.3104 | -0.2662 | -0.121 | -0.3511 | -0.4845* | -0.3435* | -0.6857** |
|  | [0.165] |  | [0.1711] | [0.1516] | [0.1258] | [0.3964] | [0.2439] | [0.1330] | [0.2374] |
| _cons | 1.118 | _cons | 0.311 | 0.8372 | 1.1304 | -3.0991 | 0.6686 | 1.3434 | 0.3924 |
|  | [1.164] |  | [0.8606] | [0.7499] | [0.6593] | [3.3679] | [1.2368] | [0.9707] | [1.1931] |
|  |  |  |  |  |  |  |  |  |  |
| N. Observations | 149 | N. Observations | 151 | 151 | 151 | 151 | 151 | 151 | 151 |
|  |  | R-/ Pseudo R-squared | 0.37 | 0.37 | 0.22 | 0.17 | 0.21 | 0.22 | 0.22 |
|  |  | Test H0: spend coef=q50 | |  |  |  |  |  |  |
|  |  | Test H0 spend coef=q10 | |  |  |  |  |  |  |
|  |  | Test H0 spend coef=q25 | |  |  |  |  |  |  |
|  |  | Test H0 spend coef=q75 | |  |  |  |  |  |  |

Notes:

Significance levels: * for p<.05, ** for p<.01, and *** for p<.001.

**Table S5**

| **Lomas et al. [4], Claxton et al. [35]** | | **ESTIMATES** | | | | | | | |
| --- | --- | --- | --- | --- | --- | --- | --- | --- | --- |
| **PBC 10 Circulatory** | | **PBC 10 Circulatory** | | | | | | | |
| 2012/13 spend | | 2012/13 spend | | | | | | | |
| SYLLR 2012/13/14 | | SYLLR 2012/13/14 | | | | | | | |
| instrument spend | | instrument spend | | | | | | | |
| weighted |  |  | unweighted | weighted | Quantile Regression | | | | |
| IV second stage (GMM) | |  | IV second stage (GMM) | |  |  |  |  |  |
| LA-level |  | PCT-level |  |  |  |  |  |  |  |
|  | mean |  | mean | mean | q50 | q10 | q25 | q75 | q90 |
| lLAg10_1213pheadOHP | -1.464*** | lg10_1213netpoppheadOHP | -1.4678*** | -1.4941*** | -1.4475*** | -0.9682** | -1.3082*** | -1.5593*** | -1.7806*** |
|  | [0.268] |  | [0.3261] | [0.2906] | [0.2039] | [0.3221] | [0.2423] | [0.1360] | [0.1722] |
| lLACARANneed1213 | 2.304*** | lCARANneed | 1.0093*** | 0.8137*** | 0.9480*** | 1.1132*** | 0.9531*** | 1.0172*** | 0.8720*** |
|  | [0.234] |  | [0.1596] | [0.1811] | [0.0873] | [0.1572] | [0.0828] | [0.0856] | [0.1098] |
| _cons | 11.541*** | _cons | 11.5613*** | 11.6850*** | 11.4676*** | 9.0168*** | 10.7230*** | 12.0688*** | 13.1948*** |
|  | [1.302] |  | [1.5853] | [1.4111] | [0.9852] | [1.5736] | [1.1873] | [0.6653] | [0.8405] |
|  |  |  |  |  |  |  |  |  |  |
| N. Observations | 149 | N. Observations | 151 | 151 | 151 | 151 | 151 | 151 | 151 |
|  |  | Pseudo R2 |  |  | 0.52 | 0.43 | 0.49 | 0.55 | 0.51 |
|  |  | Endogeneity test | 35.81 | 33.28 |  |  |  |  |  |
|  |  | Endogeneity p-value | 0.00 | 0.00 |  |  |  |  |  |
|  |  | Hansen J test | 1.26 | 0.99 |  |  |  |  |  |
|  |  | Hansen J p-value | 0.26 | 0.32 |  |  |  |  |  |
|  |  | Test H0: spend coef=q50 | |  |  |  |  |  | * |
|  |  | Test H0 spend coef=q10 | |  |  |  |  | * | * |
|  |  | Test H0 spend coef=q25 | |  |  |  |  |  |  |
|  |  | Test H0 spend coef=q75 | |  |  |  |  |  |  |

Notes:

Significance levels: * for p<.05, ** for p<.01, and *** for p<.001.

Instruments: lIncomeScale, lIMD2010

**Table S6**

| **Lomas et al. [4], Claxton et al. [35]** | | **ESTIMATES** | | | | | | | |
| --- | --- | --- | --- | --- | --- | --- | --- | --- | --- |
| **PBC 11 Respiratory** | | **PBC 11 Respiratory** | | | | | | | |
| 2012/13 spend | | 2012/13 spend | | | | | | | |
| SYLLR 2012/13/14 | | SYLLR 2012/13/14 | | | | | | | |
| instrument spend | | instrument spend | | | | | | | |
| weighted |  |  | unweighted | weighted |  |  |  |  |  |
| IV second stage (GMM) | |  | IV second stage (GMM) | | Quantile Regression | | | |  |
| LA-level |  | PCT-level |  |  |  |  |  |  |  |
|  | mean |  | mean | mean | q50 | q10 | q25 | q75 | q90 |
| lLAg11_1213pheadOHP | -1.704*** | lg11_1213netpoppheadOHP | -1.6957* | -2.1179* | -1.5856* | -0.9237 | -1.439 | -2.1543*** | -1.3774** |
|  | [0.459] |  | [0.7284] | [0.8318] | [0.7186] | [1.0632] | [0.7364] | [0.5265] | [0.4438] |
| LPERMSICK11 | 6.265*** | lPERMDISAB | 6.2950*** | 6.8531*** | 5.6388** | 5.3822* | 5.3409** | 7.1804*** | 5.5687*** |
|  | [1.189] |  | [1.6839] | [1.8566] | [1.8999] | [2.5876] | [1.6805] | [1.9052] | [1.0904] |
| LPERMSICK11SQ | 0.742*** | lPERMDISABSQ | 0.8408** | 0.9298** | 0.7391* | 0.7091 | 0.6885** | 0.9847** | 0.7289*** |
|  | [0.166] |  | [0.2586] | [0.2864] | [0.2963] | [0.3917] | [0.2599] | [0.2996] | [0.1672] |
| _cons | 23.203*** | _cons | 22.2196*** | 24.9720*** | 20.6811*** | 16.9491 | 19.4580** | 25.7621*** | 19.8558*** |
|  | [3.903] |  | [5.6845] | [6.2601] | [5.5990] | [8.9492] | [5.8788] | [4.6862] | [3.4653] |
|  |  |  |  |  |  |  |  |  |  |
| N. Observations | 149 | N. Observations | 151 | 151 | 151 | 151 | 151 | 151 | 151 |
|  |  | Pseudo R2 |  |  | 0.45 | 0.4 | 0.4 | 0.45 | 0.49 |
|  |  | Endogeneity test | 10.59 | 12.32 |  |  |  |  |  |
|  |  | Endogeneity p-value | 0.001 | 0.00 |  |  |  |  |  |
|  |  | Hansen J test | 4.49 | 4.13 |  |  |  |  |  |
|  |  | Hansen J p-value | 0.11 | 0.13 |  |  |  |  |  |
|  |  | Test H0: spend coef=q50 | |  |  |  |  |  |  |
|  |  | Test H0 spend coef=q10 | |  |  |  |  |  |  |
|  |  | Test H0 spend coef=q25 | |  |  |  |  |  |  |
|  |  | Test H0 spend coef=q75 | |  |  |  |  |  |  |

Notes:

Significance levels: * for p<.05, ** for p<.01, and *** for p<.001.

Instruments: lIncomeScale, lFTSTUDEN, lOWNOCC

**Table S7**

| **Lomas et al. [4], Claxton et al. [35]** | | **ESTIMATES** | | | | | | | |
| --- | --- | --- | --- | --- | --- | --- | --- | --- | --- |
| **PBC 13 Gastrointestinal** | | **PBC 13 Gastrointestinal** | | | | | | | |
| 2012/13 spend | | 2012/13 spend | | | | | | | |
| SYLLR 2012/13/14 | | SYLLR 2012/13/14 | | | | | | | |
| instrument spend | | instrument spend | | | | | | | |
| weighted |  |  | unweighted | weighted |  |  |  |  |  |
| IV second stage (GMM) | |  | IV second stage (GMM) | | Quantile Regression | | | | |
| LA-level |  | PCT-level |  |  |  |  |  |  |  |
|  | mean |  | mean | mean | q50 | q10 | q25 | q75 | q90 |
| lLAg13_1213pheadOHP | -1.904** | lg13_1213netpoppheadOHP | -1.6963** | -2.4014** | -1.3696* | -1.2347 | -0.8539 | -1.5829 | -1.6361 |
|  | [0.897] |  | [0.6408] | [0.8588] | [0.6180] | [1.0271] | [0.6219] | [0.9133] | [0.8948] |
| lLACARANneed1213 | 3.878*** | lCARANneed | 2.0598*** | 1.8804*** | 2.1290*** | 1.7594*** | 2.0812*** | 2.2484*** | 1.9830*** |
|  | [0.832] |  | [0.2092] | [0.2634] | [0.1862] | [0.3444] | [0.2632] | [0.1834] | [0.2228] |
| lLACARANneed1213SQ | 3.735*** | lCARANneedSQ | 3.8617** | 5.1474*** | 4.4194** | 3.2343 | 2.9991 | 4.1301* | 1.5045 |
|  | [1.352] |  | [1.2252] | [1.4761] | [1.4028] | [1.9086] | [1.8306] | [1.6899] | [1.7174] |
| _cons | 11.547*** | _cons | 10.6408*** | 13.7727*** | 9.1884** | 8.2634 | 6.6781* | 10.2801* | 10.7062** |
|  | [4.024] |  | [2.8755] | [3.8569] | [2.7585] | [4.6343] | [2.7959] | [4.0889] | [4.0108] |
|  |  |  |  |  |  |  |  |  |  |
| N. Observations | 149 | N. Observations | 151 | 151 | 151 | 151 | 151 | 151 | 151 |
|  |  | Pseudo R2 |  |  | 0.40 | 0.28 | 0.33 | 0.40 | 0.40 |
|  |  | Endogeneity test | 12.00 | 19.01 |  |  |  |  |  |
|  |  | Endogeneity p-value | 0.00 | 0.00 |  |  |  |  |  |
|  |  | Hansen J test | 4.88 | 1.13 |  |  |  |  |  |
|  |  | Hansen J p-value | 0.18 | 0.77 |  |  |  |  |  |
|  |  | Test H0: spend coef=q50 | |  |  |  |  |  |  |
|  |  | Test H0 spend coef=q10 | |  |  |  |  |  |  |
|  |  | Test H0 spend coef=q25 | |  |  |  |  |  |  |
|  |  | Test H0 spend coef=q75 | |  |  |  |  |  |  |

Notes:

Significance levels: * for p<.05, ** for p<.01, and *** for p<.001.

Instruments: lFTSTUDEN, lPOPAllLTI, lPOP16_64LTI, lIMD2010

# Health outcomes considered in DEA models

DEA depends on the adequate selection of inputs and outcomes. The NHS priority domains defined in the Five Year Forward View have been organised in five dimensions which apply to different outcome frameworks: NHS Outcome Frameworks, CCG Outcome Frameworks, and Improvement Areas (NHS England, 2014; 2015). The outcomes we use are included under these domains:

- Domain 1 - Preventing people from dying prematurely. This domain captures how successful the NHS is in reducing the number of avoidable deaths.
- Domain 2 - Enhancing quality of life for people with long-term conditions. This domain captures how successfully the NHS is supporting people with long-term conditions to live as normal a life as possible.
- Domain 3 - Helping people to recover from episodes of ill health or following injury. This domain captures how people recover from ill health or injury and, wherever possible, how it can be prevented.
- Domain 4 - Ensuring that people have a positive experience of care. This domain looks at the importance of providing a positive care experience for patients, service users and carers.
- Domain 5 - Treating and caring for people in a safe environment and protecting them from avoidable harm. This domain explores patient safety and its importance quality of care to deliver better health outcomes.

Our choice of outcomes was based on the availability of indicators under each of these domains, except Domain 5, which is not represented by the available outcomes.

Mortality data are analysed for major disease categories, including excess mortality caused by mental health problems. The Office for National Statistics (ONS) Death Registry used the ICD-10 classification of cause of death. We have selected six PBCs where the ICD-10 coverage is equal or cover a large part of the diseases included in the PBC. For infectious diseases, cancer, and circulatory diseases the mortality data covered almost 100% of the disease grouped in these PBCs. The PBC of respiratory diseases covers more diseases than the corresponding ICD-10 diseases with mortality statistics: asthma, COPD, and pneumonia. For the PBC of endocrine diseases, the only available mortality data is caused by diabetes, and this covers about 63% of mortality in the PBC. Finally, the PBC of gastrointestinal diseases in represented by mortality caused by liver disease and ulcers, covering 57% of PBC mortality.

Apart from mortality, DEA analyses considered other relevant outcomes in the clinical categories. Three main criteria were applied for the exclusion of health outcomes: (1) if there are an important number of missing data (over 20%); (2) if they are considered final outcomes. Available health outcomes were analysed, only those considered not intermediate outcomes of the healthcare sector were selected. In addition, when two or more health outcomes showed coefficients of correlation higher than 0.50, only one of the health outcomes was included. The importance of each health outcome was tested by using the Kolmogorov-Smirnov test. This test compares the efficiencies estimated when the health outcome *j* is included with the efficiency estimated when the outcome *j* is excluded. The selected health outcome corresponded to the one with the higher importance in determining the efficiency scores according to the Kolmogorov-Smirnov test.

To homogenise the variables and allow for improvement to be represented by an increase in the values, some variables' inverse were used and estimated as in equation: $Healthoutcome\_INV: =\frac{1}{\mathrm{Healthoutcome}}*100$

**Included health and healthcare outcomes**

*Endocrine*

- SYLLR_Endocrine_2014_INV: Years of life lost due to mortality from diabetes (ICD10 E10-E14). Directly age-standardised rates (DSR).
- DiabComplications_2014_INV: Indirectly age and sex standardised ratio of complications in people with diabetes. In case that the 2014 observation was missed, the most recent observation available was included.

*Cancer*

- SYLLR_Cancer_2014_INV: Years of life lost due to mortality from all cancers (ICD10 C00-C97). Directly age-standardised rates (DSR).
- OneYSurv_2014: One-year net survival for adults diagnosed with cancer (aged 15 - 99 years), 95% confidence intervals. In case that the 2014 observation was missed, the most recent observation available was included.

*Mental Health*

- SMH/CPA_Independently: Proportion of working age adults (18-69) who are receiving secondary mental health services and who are on the Care Programme Approach at the end of the month, who are recorded as living independently (with or without support) (%).
- SMH/CPA_Employment: Proportion of working age adults (18-69) who are receiving secondary mental health services and who are on the Care Programme Approach at the end of the month who are recorded as being employed (%).
- ExcessMort_2014_INV: Excess under 75 mortality rate in adults with serious mental illness. Standardised mortality ratio (SMR) expressed as a percentage based on general population and mental health population mortality rates, 95% confidence intervals (CI). In case that the 2014 observation was missed, the most recent observation available was included.
- MH_HRQoL_ 2014: Health-related quality of life for people with a long-term mental health condition. Directly standardised average health-status (EQ-5DTM) score for individuals reporting that they have a long-term mental health condition.

*Circulatory*

- SYLLR_CVD_2014_INV: Years of life lost due to mortality from all circulatory diseases (ICD10 I00-I99). Directly age-standardised rates (DSR).
- CardiacRehab_2014: Proportion of referrals to a cardiac rehabilitation programme that were recorded as completed within 365 days of the start of an associated hospital admission, expressed as a percentage. In case that the 2014 observation was missed, the most recent observation available was included.
- Stroke_discharge_2014: People with stroke who are discharged from hospital with a joint health and social care plan. In case that the 2014 observation was missed, 2015 observation was included if available.

*Respiratory*

- SYLLR_Respiratory_2014_INV: Years of life lost due to mortality from: bronchitis, emphysema and other COPD (ICD10 J40-J44); asthma (ICD10 J45-J46); and pneumonia (ICD10 J12-J18). Directly age-standardised rates (DSR).
- EmergencyChild_2014_INV: Directly age and sex standardised admission rate for emergency admissions for children aged 18 years and under with lower respiratory tract infections per 100,000 registered patients. In case that the 2014 observation was missed, the most recent observation available was included.

*Gastrointestinal*

- SYLLR_Gastro_2014_INV: Years of life lost due to mortality from: (1) gastric, duodenal and peptic ulcers (ICD10 K25-K27); and (2) chronic liver disease including cirrhosis (ICD10 K70, K73-K74).
- AlcoholLiverEmerg_2014_INV: Directly age and sex standardised rate of emergency admissions for alcohol related liver disease in adults aged 19 years and older, per 100,000 registered patients, 95% confidence intervals (CI).

*Maternity*

- NeonatalMort_2014_INV: Neonatal mortality and stillbirths. Directly age-standardised rates.
- MAT01_Point_2012: Maternity Services Quality and Outcomes Framework (QOF) for April 2009.

**Environmental variables included in the DEA models**

Two environmental variables were selected to consider the influence of those factors that affect the capacity of the PCTs to achieve higher levels of health outcomes, but PCTs' managers cannot change. First, the socio-economic situation is captured by the deprivation index. Second, the financial restrictions faced by each PCT are reflected in the difference between the budget needed to fulfill health needs and the budget allocated by the NHS. These variables cover a broad range of factors:

- Deprivation Index: The English Index of Deprivation measures relative levels of deprivation in more than 32 000 LSOAs in England. Seven domains of deprivation were considered in the 2010 estimation of the Deprivation Index: (1) income deprivation, (2) employment deprivation, (3) health deprivation and disability domain, (4) education, skills, and training deprivation, (5) barriers to housing and services, (6) crime domain, and (7) living environment deprivation.
- Distant_to_target_2010: The NHS classifies the PCTs according to the budget allocated. The budget that should be allocated to a PCT (hereafter the "required budget") is estimated based on the Need Index, which reflects the health care demand of the PCT. The Need index' formula is based on 1) the size of the registered population; 2) the need for health care services related to age and sex; 3) the unmet needs and health inequalities; 4) the unavoidably higher costs of delivering healthcare due to location alone; 5) the higher costs of providing emergency ambulance services in sparsely populated areas, and 6) the higher costs of unavoidably small hospitals with 24-hour accident and emergency services in remote areas (NHS, 2019).
  Because the allocated budget cannot be drastically changed every year, it is adjusted gradually toward the required budget. These adjustments can be positive or negative depending on if the required budget is higher or lower than the budget allocated. Therefore, we considered the difference between the allocated budget and the required budget, not the Need Index. The distance to target variables is expressed in percentage terms.

# Methodology for the DEA: three-step procedure

## First Step: Initial DEA

We followed the three-step procedure outlined by Fried et al. [24]. In the first step, DEA is applied by including all selected health outcomes and inputs, but excluding the environmental variables. In this step initial measures of PCT efficiency scores are obtained. The variable returns to scale DEA used here can be expressed as the following linear programming problem:

$${min}_{\theta, \lambda} \theta$$

*subject to* $\theta x_{i}^{q}\geq\sum_{k=1}^{K} \lambda^{k}x_{i}^{k}$

$\sum_{k=1}^{K} \lambda^{k}y_{j}^{k} \geq y_{j}^{q}$

$$\lambda^{k}\geq0$$

$\sum_{k=1}^{K} \lambda^{k}=1$ (1)

Where $x_{i}^{k}\geq0$ (*k* =1,…,K and *i*=1,…,I) is the input *i* used by PCT *k*, and $y_{j}^{k}\geq0$ is the health outcome *j* ( *j*=1,…,J ) produce by PCT *k*. $\lambda^{k}$ is the weight given to PCT *k* in its comparison with PCT *q* and $\theta$ is the efficiency score of PCT *q*. A $\theta=0$ means that PCT *q* is fully efficient. In the first step of the analysis an efficiency score is estimated for each PCT.

During this step, the DEA is estimated twice. First, all PCTs are included and a set of $\theta^{k}$ (k= 1,…,K) are estimated. Second, after the identification and exclusion of the outliers, the DEA expressed in equation (1) is re-estimated to obtain a new set of efficiencies scores that are not affected by the outliers.

## Second Step: Stochastic frontier analysis (SFA)

Fried et al. [24] assumed that inefficiencies can be obtained from the first stage by observing so-called slacks. They defined inputs slack as:

$s_{qi}=\left[ x_{i}^{q}-\sum_{k=1}^{K} \lambda^{k}x_{i}^{k} \right]$ (2)

According to Fried et al. [24] , these first estimated inefficiencies can be split into three elements: (1) environmental influences, (2) pure managerial inefficiencies, and (3) statistical noise associated to measurement errors in inputs and/or health outcomes used to generate the first stage slacks. By using equation (2) a set of slacks can be estimated for each input included. This allows the split of these three effects for each input slack, and so, to estimate the effect that environmental variables in each input slack.

If we assume that the slacks $s_{qi}$estimated in (2) are explained in part by the effect of two environmental variables, is it possible to estimate one equation for each input *i* (i=1,…,I) where environmental variables and error terms varies across K PCTs (k=1,….,K):

$s_{ki}= \beta_{i0}+\beta_{i1}{DepIndex}_{k}+\beta_{i2}{TargetDistance}_{k}+ \varepsilon_{ki}$ (3)

Where $s_{ki}$is the slack of PCT *k (k= 1,...,K)* for input *i*  (*i*=1,…,I).

In addition to the effect of the environmental variables, Fried et al. [24] suggest to take advantage of the particularities of the SFA to be able of splitting the error term $\varepsilon_{ki}$ into two elements: the statistical noise and the inefficiency. SFA is a parametric approach used to estimate production or cost functions, while explicitly accounting for the presence of producers’ inefficiency. The SFA assume that in case of inefficiency in the production, the error term $\varepsilon_{i}$ estimated in (3) is actually a composed error reflecting two elements $v_{ki} \mathrm{and} u_{ki}$ .

The first element, $v_{ki}$, reflects statistical noise and is distributed as $v_{ki}\sim N\left( 0,\sigma_{vi}^{2} \right)$; while the second one, $u_{ki},$reflects managerial inefficiency and is distributed as $u_{ki}\sim N_{+}\left( 0,\sigma_{ui}^{2} \right)$. If we assume that $v_{ki} \mathrm{and} u_{ki}$ are distributed independently of each other and of the environmental variables, we can estimated I regressions (*i*=1 …,I) using maximum likelihood techniques. In the analysis presenting in this analysis, two environmental variables are considered, therefore the equations to be estimated are:

$s_{ki}= \beta_{i0}+\beta_{i1}{DepIndex}_{k}+\beta_{i2}{TargetDistance}_{k}+v_{ki}-u_{ki}$ (4)

The minimum slack that can be achieve in a noise environment, characterized by variables $({DepIndex}_{k},{TargetDistance}_{k},v_{ki})$ and parameters $\left( \beta_{i0},\beta_{i1}{,\beta}_{i2},\sigma_{vi}^{2} \right)$, corresponds to the first part of equation (4): $(\beta_{i1}{DepIndex}_{k}+\beta_{i2}{TargetDistance}_{k}+v_{ki})$. Any slacks in excess are attributed to managerial inefficiencies, and captured by the nonnegative error component $u_{ki}$, with parameters $\left( \mu_{i},\sigma_{ui}^{2} \right)$, that reflects the variability of managerial inefficiencies across producers and inputs.

A first estimation of the SFAs expressed in equation (4) was done. Tests for the normally of the errors, heteroscedasticity and multicollinearity were considered. The Breusch-Pagan test and the Goldfeld-Quandt test indicated that statistically significant heteroscedasticity was presented in each one of the SFA estimated. When heteroscedasticity exists, correcting for it leads not only to a substantial improvement of the statistical properties of estimators but also to improved efficiency and ranking measures (Guermat et al. [36]). Therefore, instead of the original input slacks, a log transformation of $s_{ki}$ is used to estimate the SFA.

${ln(s}_{ki})= \beta_{i0}+\beta_{i1}{DepIndex}_{k}+\beta_{i2}{TargetDistance}_{k}+v_{ki}-u_{ki}$ (5)

Once equation (5) is estimated by applying the SFA, the results are used to adjust PCTs´ inputs, such that it allows for “levelling the playing field” in which the PCTs efficiencies are estimated. In this way, those PCTs benefiting from the environmental conditions will not, for this reason, appear as having higher levels of efficiency.

It is important to highlight that original inputs slacks can be expressed as:

$s_{ki}=e^{\beta_{i0}}*e^{\beta_{i1}{DepIndex}_{k}}*e^{\beta_{i2}{TargetDistance}_{k}}*e^{v_{ki}}*e^{-u_{ki}}$ (6)

Those PCTs that are in an advantaged position because of a relatively favourable environment or because a relatively better luck (represented by the statistical noise), will have their inputs adjusted upward in a proportion that represents their level of advantage. In order to estimate this proportion, the PCT that is in the worst situation is identified. The differences between the most disadvantage PCT and all others PCTs are estimated for each element:

${{ADDepIndex}_{ik}= max}_{k} \left[ e^{\beta_{i1}{DepIndex}_{k}} \right]- e^{\beta_{i1}{DepIndex}_{k}}$ (7)

${{ADTargetDistance}_{ik}= max}_{k} \left[ e^{\beta_{i2}{TargetDistance}_{k}} \right]-e^{\beta_{i2}{TargetDistance}_{k}}$ (8)

${{ADRandomNoise}_{ik}= max}_{k} \left[ e^{v_{ki}} \right]- e^{v_{ki}}$ (9)

with *k* = 1,…,K and *i* = 1,…,I

In order to estimate equation (9) ii is necessary to separate $\varepsilon_{ki}$ of equation (3) into two composed elements: statistical noise and managerial inefficiencies. From the conditional estimators for managerial inefficiency, it is possible to obtain the statistical noise residual by considering:

$\hat{E}\left[ {v_{ki}\mid v}_{ki}-u_{ki} \right]={ln(s}_{ki})- \beta_{i0}-\beta_{i1}{DepIndex}_{k}-\beta_{i2}{TargetDistance}_{k}-\hat{E}\left[ {u_{ki}\mid v}_{ki}-u_{ki} \right]$ (10)

This provides conditional (on $v_{ki}-u_{ki}$) estimators of $v_{ki}$. According to Bogetoft and Otto [23], it is possible to estimate:

$\hat{E}\left[ {u_{ki}\mid v}_{ki}-u_{ki} \right]$ =$\mu_{*}+\sigma_{*}\frac{\phi(\frac{\mu_{*}}{\sigma_{*}})}{Ф\left( \frac{\mu_{*}}{\sigma_{*}} \right)}$

Where $\delta_{i}=\sqrt{\frac{\sigma_{ui}^{2}}{\sigma_{vi}^{2}}}$

$\mu_{*}$ = $-\varepsilon_{ki}\frac{{\delta_{i}}^{2}}{(1+{\delta_{i}}^{2})}$

$\sigma_{*}=\frac{\delta_{i}}{(1+{\delta_{i}}^{2})}\sigma^{2}$ (11)

With $\varepsilon_{ki}$ distributed as $\varepsilon_{ki}\sim N\left( 0,\sigma_{i}^{2} \right)$.

Equations (7), (8) and (9) show the differences between the PCTs in terms of environment and “lucky” conditions that need to be adjusted in order to have the field levelled as the level of the most disadvantage PCT. With this in mind, adjusted inputs are estimated as follow:

${xAD}_{i}^{k}= x_{i}^{k}+{ADDepIndex}_{ik}+{ADTargetDistance}_{ik}+{ADRandomNoise}_{ik}$ (12)

## Third Step: Adjusted DEA

In the last step, ${xAD}_{i}^{k}$ are used to estimate a new set of efficiencies scores (${\theta AD}_{k}$), using the following linear programming problem:

$${min}_{\theta AD, \lambda AD} \theta$$

*subject to* $\theta ADx_{i}^{q}\geq\sum_{k=1}^{K} {\lambda AD}^{k}{xAD}_{i}^{k}$

$\sum_{k=1}^{K} {\lambda AD}^{k}y_{j}^{k} \geq y_{j}^{q}$

$${\lambda Ad}^{k}\geq0$$

$\sum_{k=1}^{K} {\lambda AD}^{k}=1$ (13)

# Efficiency Scores

Table S8. Efficiency Scores - DEA

| **PCT code** | **PCT name** | **#**  **Efficient PBCs** | **Mental Health** | **Maternity** | **Cancer** | **Gastrointestinal** | **Cardiovascular** | **Respiratory** | **Endocrine** |
| --- | --- | --- | --- | --- | --- | --- | --- | --- | --- |
| 5A3 | South Gloucestershire PCT | 5 |  | **1.00** | 1.00 | **1.00** | **1.00** | **1.00** | **1.00** |
| 5A4 | Havering PCT | 2 | **1.00** | 0.98 | 0.94 | 0.98 | 0.96 | 0.96 | **1.00** |
| 5A5 | Kingston PCT | 2 | **1.00** | 1.00 | **1.00** | 0.97 | 0.97 | 0.95 | 0.94 |
| 5A7 | Bromley PCT | 2 | 0.96 | 0.95 | 0.95 | 0.97 | **1.00** | **1.00** | 0.93 |
| 5A8 | Greenwich Teaching PCT | 3 | 0.91 | 0.85 | **1.00** | 1.00 |  | **1.00** | **1.00** |
| 5A9 | Barnet PCT | 5 | 0.92 | **1.00** | **1.00** | **1.00** | **1.00** | **1.00** | 0.95 |
| 5AT | Hillingdon PCT | 2 | **1.00** | 0.97 | 0.97 | 0.98 | **1.00** | 1.00 | 0.97 |
| 5C1 | Enfield PCT | 4 | 0.92 | 0.99 | **1.00** | **1.00** | **1.00** | **1.00** | 0.98 |
| 5C2 | Barking and Dagenham PCT | 1 | 0.97 | 0.91 | **1.00** | 0.96 | 0.95 | 0.94 | 0.96 |
| 5C3 | City and Hackney Teaching PCT | 3 | **1.00** | 0.90 | **1.00** |  | **1.00** |  | 0.95 |
| 5C4 | Tower Hamlets PCT | 2 | **1.00** | 0.82 | 0.97 | 0.93 |  | **1.00** | 0.89 |
| 5C5 | Newham PCT | 5 | **1.00** | 0.83 | **1.00** | **1.00** | **1.00** | **1.00** | 0.90 |
| 5C9 | Haringey Teaching PCT | 3 | 0.83 | 0.89 | **1.00** | **1.00** | **1.00** |  | 0.96 |
| 5CN | Herefordshire PCT | 3 | **1.00** | 0.97 | **1.00** | 0.98 |  | **1.00** | 0.98 |
| 5CQ | Milton Keynes PCT | 0 | 0.97 | 0.99 | 0.95 | 0.98 | 0.96 | 0.96 | 0.98 |
| 5D7 | Newcastle PCT | 0 | 0.91 |  | 0.95 | 0.96 |  | 0.94 | 0.99 |
| 5D8 | North Tyneside PCT | 1 | 0.98 | **1.00** | 0.96 | 0.98 |  | 0.96 | 0.98 |
| 5D9 | Hartlepool PCT | 1 | 1.00 | **1.00** | 0.98 | 0.97 |  | 0.93 |  |
| 5E1 | Stockton-on-Tees Teaching PCT | 1 | **1.00** | 0.97 | 0.92 | 0.97 |  |  | 0.99 |
| 5EF | North Lincolnshire PCT | 1 | 0.98 | 0.99 | 0.94 | 0.98 | **1.00** | 0.97 | 0.98 |
| 5EM | Nottingham City PCT | 3 | 0.96 | 0.92 | 0.93 | **1.00** |  | **1.00** | **1.00** |
| 5ET | Bassetlaw PCT | 2 | **1.00** | 0.99 | 0.96 | 0.98 | 1.00 | **1.00** | 0.98 |
| 5F1 | Plymouth Teaching PCT | 1 | 0.96 | 0.96 | 0.95 | 0.99 | 0.96 | 0.98 | **1.00** |
| 5F5 | Salford PCT | 0 | 0.91 | 0.94 | 0.95 | 0.95 | 0.97 | 0.93 | 0.96 |
| 5F7 | Stockport PCT | 2 |  |  | 0.99 | **1.00** | 0.96 | **1.00** |  |
| 5FE | Portsmouth City Teaching PCT | 1 | 0.95 | 0.94 | 0.99 | 0.97 | 0.95 | 0.96 | **1.00** |
| 5FL | Bath and North East Somerset PCT | 3 | 0.97 | **1.00** | 0.95 | 0.98 | **1.00** | **1.00** | 0.96 |
| 5GC | Luton Teaching PCT | 1 | 0.92 | 0.93 | **1.00** | 0.97 | 0.95 | 0.95 | 0.97 |
| 5H1 | Hammersmith and Fulham PCT | 1 | 0.98 | 0.94 | 0.99 | 0.97 | **1.00** | 0.95 | 0.97 |
| 5H8 | Rotherham PCT | 1 | **1.00** | 0.96 | 0.95 | 1.00 | 0.96 | 0.98 |  |
| 5HG | Ashton, Leigh and Wigan PCT | 0 | 0.99 | 0.97 | 0.94 | 0.96 | 0.95 | 0.96 | 0.97 |
| 5HP | Blackpool PCT | 0 | 0.86 | 0.96 | 0.97 | 0.98 | 0.94 | 0.89 | 0.96 |
| 5HQ | Bolton PCT | 6 |  | **1.00** | **1.00** | **1.00** | **1.00** | **1.00** | **1.00** |
| 5HX | Ealing PCT | 2 | 0.98 | 0.94 | **1.00** | 0.96 | **1.00** | 0.98 | 0.95 |
| 5HY | Hounslow PCT | 1 | 0.97 | 0.94 |  | 0.99 | **1.00** | 0.99 | 0.96 |
| 5J2 | Warrington PCT | 0 | 0.95 | 0.96 | 0.96 | 0.98 | 0.95 | 0.96 | 0.97 |
| 5J4 | Knowsley PCT | 1 | 0.91 | 0.93 | **1.00** | 0.93 | 0.95 | 0.86 | 0.93 |
| 5J5 | Oldham PCT | 0 | 0.93 | 0.94 | 0.96 | 0.98 | 0.94 | 0.90 | 0.95 |
| 5J6 | Calderdale PCT | 0 | 0.93 | 0.93 | 0.95 | 0.96 |  | 0.93 | 0.97 |
| 5J9 | Darlington PCT | 0 | 0.90 | 0.93 | 0.97 | 0.96 |  | 0.92 | 0.98 |
| 5JE | Barnsley PCT | 0 | 0.93 | 0.98 | 0.93 | 0.97 |  | 0.95 | 0.99 |
| 5JX | Bury PCT | 6 | **1.00** | **1.00** | **1.00** | **1.00** | 0.96 | **1.00** | **1.00** |
| 5K3 | Swindon PCT | 5 | **1.00** | **1.00** | 0.98 | **1.00** | **1.00** | **1.00** | 0.99 |
| 5K5 | Brent Teaching PCT | 0 | 0.95 | 0.90 | 0.96 | 0.96 | 0.94 | 0.96 | 0.92 |
| 5K6 | Harrow PCT | 1 | 0.96 | 0.95 | **1.00** | 0.98 | 0.96 | 0.97 | 0.95 |
| 5K7 | Camden PCT | 1 | 0.85 | 0.93 | 0.98 | 0.96 | **1.00** | 0.99 | 0.94 |
| 5K8 | Islington PCT | 3 | 0.75 | 0.88 | **1.00** | 0.99 | **1.00** | **1.00** | 0.91 |
| 5K9 | Croydon PCT | 3 | 0.94 | 0.92 | **1.00** | 0.96 | **1.00** | **1.00** | 0.98 |
| 5KF | Gateshead PCT | 1 | 0.95 | 0.95 | **1.00** | 0.96 |  | 0.94 | 0.98 |
| 5KG | South Tyneside PCT | 0 | 0.98 | 0.99 | 0.95 | 0.99 | 0.96 | 0.97 | 1.00 |
| 5KL | Sunderland Teaching PCT | 2 | 0.99 | 0.98 | **1.00** | 1.00 |  | 0.98 | **1.00** |
| 5KM | Middlesbrough PCT | 0 | 0.91 | 0.91 | 0.96 | 0.96 |  | 0.92 | 0.97 |
| 5L1 | Southampton City PCT | 4 | 0.96 | **1.00** | **1.00** | **1.00** | **1.00** | 0.96 |  |
| 5L3 | Medway Teaching PCT | 0 | 0.95 | 0.95 | 0.95 | 0.97 |  | 0.97 | 0.96 |
| 5LA | Kensington and Chelsea PCT | 4 | 0.78 | 0.86 | **1.00** | **1.00** | **1.00** | **1.00** | 0.91 |
| 5LC | Westminster PCT | 2 | 0.85 | 0.85 | **1.00** | 0.97 | 0.95 | **1.00** | 0.90 |
| 5LD | Lambeth PCT | 1 | 0.76 | 0.79 | **1.00** | 0.93 |  | 0.98 | 0.93 |
| 5LE | Southwark PCT | 0 | 0.90 | 0.91 |  | 0.97 |  | 0.99 | 0.98 |
| 5LF | Lewisham PCT | 4 | **1.00** | 0.99 | 0.99 | **1.00** | **1.00** | **1.00** | 0.99 |
| 5LG | Wandsworth PCT | 2 | 0.98 | 0.92 | **1.00** | 0.99 | **1.00** | 0.98 | 0.94 |
| 5LH | Tameside and Glossop PCT | 5 | **1.00** | **1.00** | 0.95 | **1.00** | 0.95 | **1.00** | **1.00** |
| 5LQ | Brighton and Hove City PCT | 6 | **1.00** | **1.00** | 0.97 | **1.00** | **1.00** | **1.00** | **1.00** |
| 5M1 | South Birmingham PCT | 2 | 0.93 | 0.96 | 0.96 | **1.00** | 0.98 | **1.00** | 0.98 |
| 5M2 | Shropshire County PCT | 4 | **1.00** |  | 0.95 | **1.00** | 0.96 | **1.00** | **1.00** |
| 5M3 | Walsall Teaching PCT | 0 | 0.93 | 0.92 | 0.94 | 0.96 | 0.96 | 0.92 | 0.95 |
| 5M6 | Richmond and Twickenham PCT | 5 | **1.00** | 0.91 | **1.00** | 0.98 | **1.00** | **1.00** | **1.00** |
| 5M7 | Sutton and Merton PCT | 3 | **1.00** | 0.96 | **1.00** | 0.98 | **1.00** | 0.97 | 0.99 |
| 5M8 | North Somerset PCT | 3 | **1.00** | 0.98 | 0.94 | 0.99 | 0.96 | **1.00** | **1.00** |
| 5MD | Coventry Teaching PCT | 1 | 0.92 | 0.92 | 0.93 | 0.98 | **1.00** | 0.98 | 0.96 |
| 5MK | Telford and Wrekin PCT | 2 | 0.95 | 0.95 | 0.93 | 0.97 | **1.00** | **1.00** | 0.98 |
| 5MV | Wolverhampton City PCT | 0 | 0.97 | 0.94 | 0.94 | 0.99 | 0.99 | 0.99 | 0.96 |
| 5MX | Heart of Birmingham Teaching PCT | 0 | 0.80 | 0.83 | 0.97 |  | 0.94 | 0.97 | 0.87 |
| 5N1 | Leeds PCT | 3 | **1.00** | **1.00** | 0.94 | 0.99 |  | **1.00** | 0.99 |
| 5N2 | Kirklees PCT | 0 | 0.97 | 0.95 | 0.96 | 0.96 | 0.95 | 0.95 | 0.96 |
| 5N3 | Wakefield District PCT | 0 | 0.93 | 0.95 | 0.93 | 0.96 | 0.95 | 0.92 | 0.97 |
| 5N4 | Sheffield PCT | 0 | 0.95 | 0.98 | 0.94 | 0.97 |  | 0.97 | 0.99 |
| 5N5 | Doncaster PCT | 1 | 0.90 | 0.96 | 0.94 | 0.98 |  | 0.94 | **1.00** |
| 5N6 | Derbyshire County PCT | 2 | **1.00** | 1.00 | 0.92 | 0.98 | **1.00** | 0.96 | 0.99 |
| 5N7 | Derby City PCT | 4 | **1.00** | 0.99 | **1.00** | **1.00** |  |  | **1.00** |
| 5N8 | Nottinghamshire County PCT | 1 | 0.98 | **1.00** | 0.94 | 0.98 | 0.98 | 0.98 | 0.98 |
| 5N9 | Lincolnshire PCT | 1 | 0.96 | **1.00** | 0.91 | 0.98 | 0.95 | 0.96 | 0.99 |
| 5NA | Redbridge PCT | 3 | **1.00** | 1.00 |  | **1.00** | 0.95 | **1.00** | 0.99 |
| 5NC | Waltham Forest PCT | 3 | **1.00** | 0.97 | **1.00** | 0.99 | **1.00** | 0.98 | 0.97 |
| 5ND | County Durham PCT | 2 | 0.96 | **1.00** | 1.00 | 0.98 | **1.00** | 0.96 | 0.99 |
| 5NE | Cumbria PCT | 2 | 0.97 | **1.00** | 0.95 | 0.98 | 0.96 | 0.96 | **1.00** |
| 5NF | North Lancashire PCT | 3 | **1.00** | **1.00** | 0.93 | **1.00** | 0.96 | 0.98 | 0.98 |
| 5NG | Central Lancashire PCT | 0 | 0.98 | 0.97 | 0.97 | 0.98 | 0.96 | 0.96 | 0.98 |
| 5NH | East Lancashire PCT | 0 | 0.91 | 0.93 | 0.95 | 0.96 | 0.96 | 0.91 | 0.95 |
| 5NJ | Sefton PCT | 1 | 0.95 | **1.00** | 0.93 | 0.95 | 0.95 | 0.93 | 0.95 |
| 5NK | Wirral PCT | 0 | 0.92 | 0.94 | 0.95 | 0.95 | 0.95 | 0.94 | 0.94 |
| 5NL | Liverpool PCT | 1 | 0.91 | **1.00** | 0.97 | 0.95 | 0.95 | 0.91 | 0.93 |
| 5NM | Halton and St Helens PCT | 0 | 0.92 | 0.95 | 1.00 | 0.95 | 0.98 | 0.88 | 0.96 |
| 5NN | Western Cheshire PCT | 0 | 0.93 | 0.99 | 0.94 | 0.97 | 0.95 | 0.96 | 0.96 |
| 5NP | Central and Eastern Cheshire PCT | 5 | **1.00** | **1.00** | **1.00** | 0.99 | 0.99 | **1.00** | **1.00** |
| 5NQ | Heywood, Middleton and Rochdale PCT | 0 | 0.91 | 0.93 | 0.95 | 0.98 | 0.95 | 0.96 | 0.95 |
| 5NR | Trafford PCT | 0 | 0.98 | 0.95 | 0.95 | 0.97 | 0.97 | 0.95 | 0.96 |
| 5NT | Manchester PCT | 1 | 0.91 | 0.96 | 0.99 | 0.96 | **1.00** | 0.94 | 0.95 |
| 5NV | North Yorkshire and York PCT | 7 | **1.00** | **1.00** | **1.00** | **1.00** | **1.00** | **1.00** | **1.00** |
| 5NW | East Riding of Yorkshire PCT | 4 |  |  | **1.00** | **1.00** | 0.96 | **1.00** | **1.00** |
| 5NX | Hull PCT | 0 | 0.93 | 0.92 | 0.94 | 0.95 | 0.97 | 0.92 | 0.97 |
| 5NY | Bradford and Airedale PCT | 0 | 0.91 | 0.91 | 0.94 | 0.94 |  | 0.94 | 0.94 |
| 5P1 | South East Essex PCT | 7 | **1.00** | **1.00** | **1.00** | **1.00** | **1.00** | **1.00** | **1.00** |
| 5P2 | Bedfordshire PCT | 3 | **1.00** | **1.00** | 0.95 | 0.98 | **1.00** | 0.98 | 0.98 |
| 5P5 | Surrey PCT | 1 | 0.96 | **1.00** | 0.99 | 0.98 | 0.98 | 0.97 | 0.96 |
| 5P6 | West Sussex PCT | 1 | 0.96 | 0.98 | 0.98 | 0.98 | **1.00** | 0.96 | 0.96 |
| 5P7 | East Sussex Downs and Weald PCT | 4 | 0.96 | **1.00** | 0.93 | **1.00** | **1.00** | 0.95 | **1.00** |
| 5P8 | Hastings and Rother PCT | 1 | 0.95 | **1.00** | 0.93 | 0.95 | 0.98 | 0.93 | 0.95 |
| 5P9 | West Kent PCT | 3 | **1.00** | **1.00** | **1.00** | 0.99 | 0.97 | 0.98 | 0.98 |
| 5PA | Leicestershire County and Rutland PCT | 4 | **1.00** | **1.00** | 0.99 | **1.00** | 0.97 | **1.00** | 0.99 |
| 5PC | Leicester City PCT | 4 | 0.95 | 0.93 | **1.00** | **1.00** | **1.00** | **1.00** | 0.99 |
| 5PD | Northamptonshire PCT | 2 | 0.93 | **1.00** | 0.98 | 0.98 | **1.00** | 0.95 | 0.97 |
| 5PE | Dudley PCT | 1 | **1.00** | 0.96 | 0.92 | 0.99 | 0.96 | 0.98 | 0.98 |
| 5PF | Sandwell PCT | 0 | 0.95 | 0.95 | 0.95 | 0.98 | 0.97 | 0.96 | 0.96 |
| 5PG | Birmingham East and North PCT | 2 | 0.91 | 0.92 | **1.00** | **1.00** |  | 0.99 | 0.95 |
| 5PH | North Staffordshire PCT | 2 | **1.00** | 0.97 | 0.93 | 0.98 | **1.00** | 0.95 | 0.97 |
| 5PJ | Stoke on Trent PCT | 2 | **1.00** | 0.93 | 0.96 | 0.99 | **1.00** | 0.92 | 0.97 |
| 5PK | South Staffordshire PCT | 3 | **1.00** | **1.00** | 0.92 | 0.98 | 0.97 | **1.00** | 0.98 |
| 5PL | Worcestershire PCT | 3 | **1.00** | 1.00 | 0.96 |  | 0.98 | **1.00** | **1.00** |
| 5PM | Warwickshire PCT | 3 | **1.00** | **1.00** | **1.00** | 0.98 | 0.98 | 0.99 | 0.98 |
| 5PN | Peterborough PCT | 1 | 0.85 | 0.91 | **1.00** | 0.96 |  | 0.92 | 0.94 |
| 5PP | Cambridgeshire PCT | 1 | 0.99 | 0.98 | 0.99 | **1.00** | 0.97 | 0.99 | 0.98 |
| 5PQ | Norfolk PCT | 4 | 0.96 | **1.00** |  | 0.99 | **1.00** | **1.00** | **1.00** |
| 5PR | Great Yarmouth and Waveney PCT | 1 | **1.00** | 0.97 | 0.94 | 0.97 |  | 0.95 | 0.99 |
| 5PT | Suffolk PCT | 6 | **1.00** | **1.00** | 0.95 | **1.00** | **1.00** | **1.00** | **1.00** |
| 5PV | West Essex PCT | 0 | 0.97 | 0.95 | 0.95 | 0.98 | 0.96 | 0.96 | 0.96 |
| 5PW | North East Essex PCT | 5 | **1.00** | 1.00 | 0.96 | **1.00** | **1.00** | **1.00** | **1.00** |
| 5PX | Mid Essex PCT | 3 | **1.00** | **1.00** | 0.95 | **1.00** |  | 1.00 | 0.98 |
| 5PY | South West Essex PCT | 0 |  | 0.96 | 0.96 | 0.98 | 0.95 | 0.96 | 0.96 |
| 5QA | Eastern and Coastal Kent PCT | 4 | 0.92 | **1.00** | **1.00** | **1.00** | 0.99 | **1.00** | 0.96 |
| 5QC | Hampshire PCT | 5 |  |  | **1.00** | **1.00** | **1.00** | **1.00** | **1.00** |
| 5QD | Buckinghamshire PCT | 5 | **1.00** | 0.98 | **1.00** | **1.00** | **1.00** | **1.00** | 0.98 |
| 5QE | Oxfordshire PCT | 3 | **1.00** | 0.98 | **1.00** | 0.99 | **1.00** | 0.99 | 1.00 |
| 5QF | Berkshire West PCT | 3 | **1.00** | 0.98 | 0.97 |  |  | **1.00** | **1.00** |
| 5QG | Berkshire East PCT | 2 | **1.00** | 0.96 | 0.99 | **1.00** | 0.98 | 0.97 | 0.97 |
| 5QH | Gloucestershire PCT | 1 | **1.00** | 0.98 | 0.96 | 0.98 | 0.96 | 0.95 | 0.98 |
| 5QJ | Bristol PCT | 4 | 0.94 | 0.96 | **1.00** | 1.00 | **1.00** | **1.00** | **1.00** |
| 5QK | Wiltshire PCT | 3 | **1.00** | 0.98 | 0.95 | 0.98 | **1.00** | **1.00** | 0.98 |
| 5QL | Somerset PCT | 1 | **1.00** | 0.97 | 0.93 | 0.98 | 0.98 | 0.97 | 0.98 |
| 5QM | Dorset PCT | 3 | 1.00 | **1.00** | 0.95 | 0.98 | **1.00** | 0.99 | **1.00** |
| 5QN | Bournemouth and Poole PCT | 1 | 0.97 | 0.96 | 0.98 | 0.99 | 0.95 | **1.00** | 0.99 |
| 5QP | Cornwall and Isles of Scilly PCT | 5 | **1.00** | **1.00** | 1.00 | **1.00** | **1.00** |  | **1.00** |
| 5QQ | Devon PCT | 0 | 0.97 | 0.98 | 0.94 | 0.99 | 0.99 | 0.99 |  |
| 5QR | Redcar and Cleveland PCT | 0 | 1.00 | 0.96 | 0.94 | 0.97 |  | 0.95 | 0.97 |
| 5QT | Isle of Wight Healthcare PCT | 4 |  | **1.00** | 0.94 | **1.00** | **1.00** | 0.99 | **1.00** |
| 5QV | Hertfordshire PCT | 6 | **1.00** | **1.00** | **1.00** | **1.00** | 0.98 | **1.00** | **1.00** |
| TAC | Northumberland Care Trust | 3 | **1.00** | **1.00** | 0.94 | 0.98 |  | 0.97 | **1.00** |
| TAK | Bexley Care Trust | 1 | 0.97 | 0.99 | **1.00** | 0.98 |  | 0.99 | 0.98 |
| TAL | Torbay Care Trust | 2 | 0.90 | **1.00** | 0.98 | 0.95 | **1.00** | 0.94 | 0.98 |
| TAM | Solihull PCT | 2 | **1.00** | 0.99 | 0.94 | **1.00** |  | 0.97 | 0.99 |
| TAN | North East Lincolnshire PCT | 0 | 0.84 | 0.92 | 0.97 | 0.92 | 0.95 | 0.85 | 0.96 |
| TAP | Blackburn with Darwen Teaching Care Trust Plus | 0 | 0.85 | 0.92 | 0.95 | 0.91 | 0.95 | 0.81 | 0.97 |

# Efficiency rankings

Table S9. Efficiency Ranking of PBCs

| **PBC** | **% of fully**  **efficient PCTs**^†^ | **Ranking**  **efficient** | **% of PCTs that can decrease expenditures in more than 5%** | **Ranking**  **Inefficient** |
| --- | --- | --- | --- | --- |
| Circulation | 43.2 | 1 | 35.8 | 7 |
| Mental health | 36.1 | 2 | 33.3 | 5 |
| Respiratory | 32.9 | 3 | 23.3 | 3 |
| Cancer | 29.9 | 4 | 31.9 | 4 |
| Maternity | 28.1 | 5 | 33.6 | 6 |
| Gastrointestinal | 26.5 | 6 | 6.8 | 1 |
| Endocrine | 23.3 | 7 | 16.4 | 2 |

†Fully efficient PCTs are those with estimated efficient score equal to 1

Note: the denominators for calculating the percentages in Table 5 are effective number of PCTs used in the estimations without counting missing data and outliners.

# Comparison of DEA and QR estimations

Table S10. Comparison DEA and QR estimations

|  | **Spearman rank correlation**† | | **Mean comparison test** | | |
| --- | --- | --- | --- | --- | --- |
|  | **No EV** | **With EV** | **Fully efficient**  **µ1** | **Non-fully efficient**  **µ0** | **p-value**  **H0: µ0=µ1**  **H1: µ0≠µ1** |
| PBC 2: Cancer | -0.211** | -0.214*** | 0.346 | 0.478 | 0.003*** |
| PBC 4: Endocrine | -0.107 | -0.078 | 0.134 | 0.248 | 0.099* |
| PBC 10: Circulation | -0.267*** | -0.322*** | 1.314 | 1.493 | 0.007*** |
| PBC 11: Respiratory | -0.169** | -0.194** | 1.264 | 1.546 | 0.009*** |
| PBC 13: Gastrointestinal | -0.230*** | -0.292*** | 1.084 | 1.366 | 0.002*** |
| † Correlation between the elasticities (QR estimations) and PCTs’ DEA efficiency scores.  Notes: With EV = correlations considering the efficiency scores estimated after adjusting by the environmental variables. No EV = correlations considering the efficiency scores without environmental factors adjustment.  Significance levels: *** p<0.01, ** p<0.05, * p<0.1 | | | | | |
